# Distinctive spore architecture and developmental biology of *Turicibacter sanguinis* reveal unexpected diversity among gut spore formers

**DOI:** 10.1128/jb.00029-26

**Published:** 2026-07-14

**Authors:** Catalina Cortés-Tapia, Francisca Cid-Rojas, Ana Moya-Beltrán, José García-Yunge, Matías Castro, Camila Rojas-Villalobos, Fernando Gil, Raquel Quatrini, Marjorie Pizarro-guajardo, Daniel Paredes-Sabja

**Affiliations:** 1 ANID – Millennium Science Initiative Program – Millennium Nucleus in the Biology of the Intestinal Microbiota 567695 , Santiago, Chile; 2 Department of Biology, Texas A&M University 14736, College Station, Texas, USA; 3 Centro Científico y Tecnológico de Excelencia Ciencia & Vida 682708, Santiago, Chile; 4 Departamento de Informática y Computación, Facultad de Ingeniería, Universidad Tecnológica Metropolitana 28092, Santiago, Chile; 5 Facultad de Ciencias, Universidad San Sebastián 373328 , Santiago, Chile; 6 Microbiota-Host Interactions & Clostridia Research Group, Center for Biomedical Research and Innovation (CIIB), School of Medicine, Faculty of Medicine, Universidad de los Andes 28060, Santiago, Chile; The Ohio State University, Columbus, Ohio, USA

**Keywords:** *Turicibacter sanguinis*, sporulation, germination, Erysipelotrichia, spore, engulfment, feeding tube

## Abstract

Sporulation is a widespread but incompletely characterized trait among gut commensals, where it underpins microbial persistence, transmission, and ecological resilience. Most insights into spore biology derive from Bacilli and Clostridia; however, little is known about sporulation in phylogenetically distant gut-associated lineages. *Turicibacter sanguinis*, a strict anaerobe linked to host serotonin metabolism, lipid homeostasis, and neurodegenerative disease, represents one such understudied taxon. Here, we integrate ultrastructural, physiological, and comparative genomic analyses to define the sporulation and germination program of *T. sanguinis*. We show that *T. sanguinis* forms heat-resistant spores with a canonical core-cortex-coat architecture but displays previously undescribed features, including a dual-layered outer envelope and bimodal electron-dense coat morphotypes. Developmental stages of sporulation follow canonical stages of *Bacillus*- and *Clostridium*-like sporulation, while genomic analyses reveal a hybrid regulatory architecture combining Clostridial-type Spo0A initiation with Bacillus-like late-stage sigma factor control. Germination assays and genomic signatures further indicate a nutrient-responsive, Bacillus-like pathway involving Ger-family receptors, SpoVA-mediated Ca-DPA release, and CwlJ- and SleM-type cortex hydrolases. Together, these findings identify *T. sanguinis* as a distinct spore-forming lineage within the human gut microbiota and expand the known diversity of sporulation strategies across the Firmicutes.

**IMPORTANCE:**

The gut bacterium *Turicibacter sanguinis* is linked to critical host functions, including serotonin production and lipid homeostasis. In this work, we show that *T. sanguinis* forms spores as a potential mechanism to survive and transmit. Unlike well-studied bacteria, *T. sanguinis* encodes a unique, hybrid sporulation program that mixes regulatory and structural elements from distant bacterial species. These observations fill a significant gap in our understanding of gut microbial ecology. It suggests that *T. sanguinis* persists in the gut through a distinct, specialized survival program. The outlined mechanisms provide a roadmap to study how this bacterium persists in the gut and impacts host health.

## INTRODUCTION

The human gastrointestinal (GI) tract is populated with a highly diverse community of microbes (1, 2). Nearly 2,000 bacterial species have been identified in the human gut, and it is estimated that the gut microbiota contains nearly 150 times more genes than the human genome (3, 4). This large genetic and metabolic potential of the gut microbiome underpins its ubiquity in nearly all aspects of human biology, including health maintenance, development, aging, and disease (5).

Throughout an individual’s lifespan, the gut microbial community is subjected to multiple episodes of dysbiosis with profound implications in its health-related roles, including the resistance to infectious diseases, metabolic balance, cognitive decline, aging, and neurodegenerative conditions such as Alzheimer’s disease (AD), further emphasizing the role of the microbiome (6–9). The most common causes of external perturbations to the microbiome are diet, medications (e.g., antibiotics), and environmental exposures (10, 11).

Recovery from these disruptions depends not only on the cessation of the perturbing factor but also on the ability of commensal microbes to recolonize the gut and re-establish functional community networks (11). Recolonization is therefore a critical determinant of both short- and long-term outcomes of microbiome disturbance. Importantly, nearly 40% of gut commensal genera are capable of forming spores (12), a feature that allows them to persist outside the host (13), withstand environmental stressors, and efficiently reseed the gut following perturbation (14). This widespread capacity for sporulation positions spore-forming commensals as key mediators of microbiome resilience and stability.

Among the spore-forming commensals, *Turicibacter sanguinis*, a gram-positive member of the Turicibacteraceae family, represents a particularly intriguing component of the gut microbiota. This bacterium harbors a functional serotonin transporter and metabolizes gut serotonin (15) and likely modulates gut-serotonin levels with potential implications in the gut-brain axis. Notably, *Turicibacter* abundance has been reported to increase in individuals with Alzheimer’s disease (16–20), further implicating it in the interplay between dysbiosis and neurodegeneration. Moreover, extensive studies have linked *T. sanguinis* with alterations of the host’s bile acid metabolism (15, 21–23). In addition to these unique physiological features, *T. sanguinis* forms resilient spores, which likely contribute to its persistence in the gut ecosystem and ability to recolonize following perturbations. Despite its potential role in the gut-brain axis, little is known about *T. sanguinis* basic physiology.

In this work, we focused on the characterization of *T. sanguinis* spore formation. Specifically, our results demonstrate that *T. sanguinis* clusters as a phylogenetically diverse group separated from members of the Erysipelotrichia class. *T. sanguinis* spore formation initiates after 2 days in YCFA agar plates, following canonical developmental stages similar to those of well-studied *Bacillus subtilis* and *Clostridioides difficile*. Ultrastructural analysis revealed that *T. sanguinis* spores, despite lacking an exosporium layer, had an outermost crust-like layer covering the coat-like layer, which exhibited two distinctive types (thick and thin). Additionally, these spores contained similar spore-core DPA levels *as C. difficile* spores and germinated in the presence of culture-rich medium. Genomic analysis of an assembled hybrid genome reveals that *T. sanguinis* encodes the master regulator of sporulation, Spo0A, and all four canonical sporulation-specific RNA polymerase sigma factors, with some sigma factor-specific regulators (SpoVT and GerE). Notably, the germination machinery seems to include predicted GerA-family of receptors, SpoVA DPA-channel, and CwlJ-type spore peptidoglycan cortex-specific enzymes. Overall, these results provide the first insight into spores of the *Turicibacteraceae* family and broaden our understanding of the diversity of spore formation in spores of the understudied Erysipelotrichia class.

## MATERIALS AND METHODS

### Bacterial growth conditions

Bacterial microbiota strains isolated, including *T. sanguinis* K031, were routinely grown at 37°C under anaerobic conditions (90% N_2_, 5% CO_2_, and 5% H_2_) in a Bactron EZ2 anaerobic chamber (Shellab, USA) in previously described YCFA medium (12): Casitone 10 g/L (BD, USA), yeast extract 2.5 g/L (BD, USA), NaHCO_3_ 4 g/L (Merck, USA), L-cysteine 1 g/L (Merck, USA), K_2_HPO_4_ 0.45 g/L (Merck, USA), KH_2_PO_4_ 0.45 g/L (Merck, USA), NaCl 0.9 g/L (Merck, USA), MgSO_4_ × 7H_2_O 0.09 g/L (Sigma-Aldrich, Chile), CaCl_2_ 0.09 g/L (Merck, USA), resazurin 1 mg/L (Sigma-Aldrich, Chile), hemin 10 mg/L (Sigma-Aldrich, Chile), biotin 10 μg/L (Sigma-Aldrich, Chile), cobalamin (vitamin B12) 10 μg/L (Sigma-Aldrich, Chile), p-aminobenzoic acid 30 μg/L (Sigma-Aldrich, Chile), folic acid 50 μg/L (Sigma-Aldrich, Chile), and pyridoxamine 150 μg/L (Sigma-Aldrich, Chile). After autoclaving (121°C, 15 min), supplemented with sodium acetate 33 mM (Sigma-Aldrich, Chile), sodium propionate 9 mM (Sigma-Aldrich, Chile), isobutyrate 1 mM (Sigma-Aldrich, Chile), isovalerate 1 mM (Sigma-Aldrich, Chile), valerate 1 mM (Sigma-Aldrich, Chile), thiamine 0.05 μg/mL (Sigma-Aldrich, Chile), riboflavin 0.05 μg/mL (Sigma-Aldrich, Chile), maltose 0.2 g/mL (Sigma-Aldrich, Chile), cellobiose 0.1 g/mL (Sigma-Aldrich, Chile), and glucose 0.2 g/mL (Sigma-Aldrich, Chile). Stock solutions were made by dissolving their components in Milli-Q water and filter sterilizing (0.2 μm pore size) prior to use.

*C. difficile* R20291 strain was grown at 37°C under anaerobic conditions (90% N2, 5% CO_2_, and 5% H_2_) on Brain Heart Infusion supplemented with 0.5% yeast extract and 0.1% L-cysteine (BHIS) broth or 1.5% agar. *E. coli* DH5α strains were grown on Luria-Bertani supplemented with 50 μg/mL chloramphenicol, 10 μg/mL tetracycline, 50 μg/mL kanamycin, or 100 μg/mL ampicillin where indicated.

Sporulation of *B. subtilis* PY79 and AZ703 was induced by the exhaustion method (24). Briefly, after 35 h of growth in Difco Sporulation (DS) medium at 37°C with vigorous shaking, spores were collected, washed, and purified. The purification was performed using KCl 1 M, lysozyme 10 mM, NaCl 1 M, SDS 0.05%, and several washes with water.

### Bacterial isolation

Fecal samples were obtained from healthy Chilean individuals within the framework of the project Millennium Nucleus in the Biology of Intestinal Microbiota. This project was approved by Comité de Bioética de la Facultad de Ciencias de la Vida, Universidad Andrés Bello, file number 013-2017. All patients enrolled in this study agreed to participate and signed an informed consent form.

Once stool samples were collected in sterile containers without preservation media (one fecal sample per donor, minimum 0.5 g), samples were reduced in an anaerobic chamber (Bactron EZ2, ShellLab) within 1 h of passing to preserve the viability of anaerobic bacteria. Culture media, sterile phosphate-buffered saline (PBS), and all other materials used for culturing were placed in the anaerobic cabinet 24 h before use to reduce oxygen levels. The fecal samples were divided into two fractions; one part was homogenized in reduced PBS (0.1 g stool/mL PBS) and was serially diluted (10^−1^–10^−8^) and plated directly onto reduced YCFA agar plates, and incubated 72–96 h at 37°C under anaerobic conditions. The other fraction was treated with an equal volume of 70% (wt/vol) ethanol (e.g., 3 g of sample mixed with 7 mL of 100% ethanol), for 4 h at room temperature under ambient aerobic conditions to kill vegetative cells. Cells were precipitated by centrifugation (6,500 × *g*, 10 min), washed three times with PBS, and the pellet was resuspended in reduced PBS under anaerobic conditions, plated onto YCFA agar plates and incubated 24–48 h under anaerobic conditions at 37°C. Their quality and morphology were evaluated by classical microbiological techniques (macroscopic and microscopic observation). The verified colonies were propagated in YCFA broth, incubated at 37 °C for 24 h under anaerobic conditions, and finally stored in 5% DMSO (Sigma-Aldrich, Chile) at −80°C.

### DNA extraction and 16S rRNA amplification

*T. sanguinis* isolate was identified by 16S rRNA amplification and subsequent whole-genome sequencing. Genomic DNA was phenol extracted using adapted protocols (25). Briefly, 1 mL of O/N culture of each strain isolated was centrifuged at 14,000 × *g* for 5 min, the supernatant was discarded and homogenized with 12.5 μL lysozyme (10 mg/mL) (Merck) and 62.5 μL Buffer TES (Tris-HCl 50 mM (Merck), EDTA 0.5 mM (Merck), and SDS 1% (Merck) to incubate at 37°C for 30 min with dry heat; 2.5 μL of Proteinase K (20 mg/mL, Merck) and 15 μL of SDS 10% was added to the tube and incubated at 55 °C for 20 min under wet heat. Once the sample reached room temperature (22°C), 75 μL of phenol:isopropanol:chloroform (25:24:1) was added to the sample and hand swirled for 5 min before centrifugation at 14,000 × *g* for 5 min. The aqueous phase was transferred into a new Eppendorf tube, and genomic DNA was precipitated with a solution of 250 μL of ethanol and 10 μL of 2.5 M NaCl (Merck) at −20°C for 15 min. Finally, the sample was centrifuged at 14,000 × *g* for 10 min, the supernatant was discarded, and precipitated genomic DNA was resuspended in 30 μL of ultrapure water with RNase and stored at −20°C until use.

The 16S rRNA amplification was done with universal primers: 7F 5′-AGAGTTTGATYMTGGCTCAG-3′ and 1510R 5′-ACGGYTACCTTGTTACGACTT-3′. PCR reaction was prepared with 8 μL Mix 2× KAPA HiFi, 1 μL primer 7F, 1 μL primer 1510R, 1 μL genomic DNA, and 9 μL ultrapure water to a final volume of 20 μL under the following conditions: initial denaturation of 95°C for 3 min, 35 cycles of 98°C for 30 s, 60°C for 20 s, 72°C for 2 min, and a final extension of 72°C for 2 min. PCR product was purified and sequenced by Macrogen USA. The isolate of interest was named as K031.

### Illumina and PacBio DNA sequencing

Genomic DNA was extracted from a culture of *T. sanguinis* K031 using DNeasy Blood and Tissue Kit (Qiagen) following manufacturer’s recommendations with the following modifications: a pellet of an overnight culture (OD_600_ of 0.3) was resuspended in 180 μL enzymatic lysis buffer and incubated for 30 min, then 25 μg/mL of RNAase were added and incubated at 37°C for 30 min and at 56°C for 1 h. DNA was sequenced with MicrobesNG (University of Birmingham, UK) according to the manufacturer’s protocol; DNA was quantified in triplicates with the Quant-iT dsDNA HS assay in an Eppendorf AF2200 plate reader.

For Illumina short-read sequencing, genomic DNA libraries were prepared using Nextera XT Library Prep Kit (Illumina, San Diego, USA) following the manufacturer’s protocol with the following modifications: 2 ng of DNA were used as input, and PCR elongation time was increased to 1 min from 30 s. DNA quantification and library preparation were carried out on a Hamilton Microlab STAR automated liquid handling system. Pooled libraries were quantified using the KAPA Biosystems Library Quantification Kit for Illumina on a Roche LightCycler 96 qPCR machine. Libraries were sequenced on the Illumina HiSeq using a 250 bp paired-end protocol.

For PacBio sequencing, Single Molecule, Real-Time sequencing (SMRT) libraries were prepared using the SMRTbell Express Template Prep Kit 2.0 (PacBio) following the manufacturer’s protocol, including DNA damage repair, end repair, and adapter ligation. Libraries were purified with AMPure PB beads and size-selected on a BluePippin (Sage Science) with a 10–15 kb cutoff to enrich long inserts. Following library QC (Qubit and TapeStation), the sequencing primer was annealed and polymerase was bound (Sequel II/IIe Binding Kit), and complexes were loaded on a Sequel IIe instrument using SMRT Cell 8M. Data were collected with a 15–30 h movie time, and Circular Consensus Sequencing reads (“HiFi”) were generated in SMRT Link using default parameters to yield Q20+ consensus reads suitable for downstream assembly and polishing.

### *T. sanguinis* K031 genome assembly and annotation

Raw reads were preprocessed using Trimmomatic v0.36 (26) to trim adaptors and remove low-quality reads. Read quality was visualized in FastQC v0.11.8 (http://www.bioinformatics.babraham.ac.uk/projects/fastqc/). *De novo* assembly of Illumina reads was performed with Velvet v1.2.10, retaining reads with quality scores > Q30. Quality control and *de novo* assembly of PacBio long reads were conducted using Canu v2.2 (27). Hybrid assembly was then performed with SPAdes v3.14.0 (28), in which PacBio long reads were used to bridge Illumina short reads. Resulting contigs were further refined using an in-house pipeline that included manual sorting, curation, and comparative genomic analyses. Assembly integrity was validated by mapping Illumina reads back to the draft genome using Bowtie2 (29). The final genome sequence was deposited in NCBI GenBank (JAGBKO000000000) and annotated using the NCBI Prokaryotic Genome Annotation Pipeline (PGAP) (30) (Table S1A). The *dnaA* gene was designated as the first locus of the final assembly.

### Publicly available *Turicibacter* spp. Database

Additional publicly available genomes of *Turicibacter* spp. were retrieved in February 2024 using NCBI data sets command-line tools (https://www.ncbi.nlm.nih.gov/datasets/) with the parameters: taxon “*Turicibacter*” and --assembly-source “GenBank” (Table S1B). Retrieved genomes were assessed for completeness (>90%) and duplication (<5%) using BUSCO and universal ortholog analysis as described by Raes et al. (31). Assembly statistics for the curated data set of 31 genomes were summarized (Table S1C).

### Taxonomic allocation using 16S rRNA and ribosomal protein phylogenies

A total of nine 16S rRNA genes identified in the hybrid *T. sanguinis* K031 genome sequence were compared and clustered according to sequence identity (Table S2A). The taxonomic allocation of *T. sanguinis* K031 isolate was verified based on 16S rRNA gene sequence analysis. Ribosomal RNA genes were downloaded from the NCBI nucleotide database using Entrez Direct v14.6 (https://www.ncbi.nlm.nih.gov/books/NBK179288/; February 2024) with the query: “*Turicibacter* 16S ribosomal RNA.” A total of 359 sequences were extracted and filtered for >1,000 bp in length. An additional 320 sequences (>1,000 bp) were extracted from GenBank genome assemblies of the *Turicibacter* genus. This yielded a data set of 431 sequences, which were clustered at 100% identity using CD-HIT v4.8.1 (32), with minimum alignment coverage of 100%. A total of 59 representative sequences were selected for downstream taxonomic and phylogenetic analyses (Table S2B). The 16S rRNA gene of isolate K031 (Table S2A) was aligned against this curated panel using BLASTN (NCBI BLAST+; megablast; E-value ≤1 × 10⁻²⁰, identity ≥97%, query coverage ≥98%) (32, 33), and assignments were confirmed by a reciprocal BLAST in which each top subject sequence was queried back against the K031 data set; annotations were accepted only when the reciprocal best hit returned K031 with comparable identity and coverage. Because of the continuous taxonomic reclassification of the *Erysipelotrichales* order, to select the appropriate outgroups, we followed the NCBI taxonomy to the order level, and from there we chose two families, each represented by one genus and one type-strain genome with a complete genome sequence, ensuring that none were candidate taxa.

Sequences were aligned with MAFFT v7.490 using the FFT-NS-2 algorithm, and the resulting alignment was trimmed with trimAl v1.2 at a gap threshold of 0.5. Phylogenetic relationships were inferred using maximum likelihood (ML), Bayesian inference (BI), and neighbor-joining (NJ). The ML tree was constructed with PhyML v3.0 (34). The BI tree was generated using MrBayes v3.2.7, run for 10,000 generations with trees sampled every 100 generations, and posterior probabilities were calculated after discarding the first 25% of sampled trees. The NJ tree was constructed with QuickTree (35) using the UPGMA and Kimura options, and bootstrap support values were calculated from 10,000 iterations. An additional phylogenetic tree was generated with FastTree v2.1.10 (double precision) (36) and visualized with the Interactive Tree Of Life (iTOL) v4 (http://itol.embl.de). Well-supported nodes were defined as those with bootstrap values ≥ 95%.

In parallel, a concatenated set of ribosomal proteins (Table S2C) was aligned with MAFFT v7.310 using the L-INS-i method (37). Alignments were checked and refined manually. Phylogenetic trees were again reconstructed using three approaches. ML trees were generated with PhyML v3.0 (34) using Smart Model Selection (SMS v1.8.4) and 1,000 bootstrap replicates. NJ trees were built with FastME v2.0 (38) using the WAG substitution model with a gamma distribution and 1,000 bootstrap replicates. Finally, BI trees were generated with MrBayes v3.2.6 (39), run for 1,000 generations with trees sampled every 100 generations, and posterior probabilities were calculated after discarding the first 30% of trees as burn-in, using a nucmodel and MCMC (Markov chain Monte Carlo) analysis.

### Overall genome relatedness index calculations

The specific assignment of K031 (and related strains) was further validated using whole-genome relatedness indices, namely Average Nucleotide Identity (ANI) (40, 41) and digital DNA-DNA hybridization (dDDH) (42, 43) using the 31 *Turicibacter* spp. genomes (Table S1C). The overall genome relatedness indexes were calculated from all possible pairwise genome comparisons using both nucleotide- and amino acid-based metrics. Average nucleotide identity based on BLAST (ANIb) was calculated using the Python package pyani (https://github.com/widdowquinn/pyani) (41) (Table S3A); *in silico* DNA-DNA hybridization index (dDDH was calculated using the Genome-to-Genome Distance Calculator with recommended formula 2 (43) and species cutoff limits defined by Meier-Kolthoff and colleagues (42) available at http://ggdc.dsmz.de (Table S3B). Additionally, average amino acid identity (AAI) was calculated using CompareM (https://github.com/donovan-h-parks/CompareM) (Table S3C).

### Growth profile of *T. sanguinis* K031

The growth profile of *T. sanguinis* K031 was determined by inoculating 1% (vol/vol) of an overnight culture into fresh YCFA broth. Cultures were incubated anaerobically at 37 °C for 12 h. At 0, 2, 4, 5, 6, 7, 8, 9, 10, 11, and 12 h, 1 mL aliquots were collected and optical density at 600 nm (OD_600_) was measured. Non-inoculated medium served as a blank control, and these baseline values were subtracted from sample measurements. The assay was performed three independent times, each in triplicate, and the results are presented as mean ± SEM, with growth curves generated using Prism 8 software (GraphPad).

### Spore preparation and purification

*T. sanguinis* K031 spores were purified by adapting previously described *Clostridioides difficile* spore purification protocols (44, 45). Briefly, 100 μL of a 1:100 dilution of an overnight culture in YCFA broth was spread on YCFA agar plates (1.5% [wt/vol] Bacto agar; BD, USA) and incubated anaerobically at 37°C for 3 days. Colonies were scraped from plates into ice-cold sterile Milli-Q water and washed five times by centrifugation at 14,000 × *g* for 5 min at 4°C. The pellet was resuspended in 45% (wt/vol) autoclaved Nycodenz (Axell, USA) and centrifuged at 14,000 × *g* for 40 min to separate spores. The spore-containing pellet was recovered and washed five additional times with ice-cold sterile Milli-Q water (14,000 × *g*, 5 min) to remove residual Nycodenz. Finally, spores were enumerated using a Neubauer chamber, adjusted to a final concentration of 5 × 10^9^ spores/mL, and stored at −80°C.

### Transmission electron microscopy (TEM)

*T. sanguinis* spores were processed and analyzed using prior published methods (44, 45). Briefly, three samples were prepared: (i) 1 mL of overnight culture of *T. sanguinis* K031 grown in YCFA broth, (ii) 1 mL of sporulated culture grown on YCFA plates, and (iii) 100 μL of purified spores from frozen stock (5 × 10^9^ spores/mL). Samples were centrifuged at 14,000 × *g* for 5 min, the supernatant was removed, and the pellets were fixed by gently layering 500 μL of 3% glutaraldehyde in 0.1 M cacodylate buffer (pH 7.2) without resuspension. Pellets were incubated at 4 °C for at least 24 h (primary fixation), followed by postfixation in 1% osmium tetroxide in 0.1 M cacodylate buffer (pH 7.2). Samples were then washed in cacodylate buffer, stained with 1% tannic acid for 30 min, and dehydrated through an acetone gradient (30% for 30 min, 50% for 30 min, 70% overnight, 90% for 30 min, and twice in 100% for 20 min each). Dehydrated pellets were infiltrated with Spurr’s resin using graded acetone:resin ratios of 3:1, 1:1, and 1:3 (40 min each), resuspended in 100% Spurr’s resin for 4 h, and polymerized overnight at 65°C. Ultrathin sections (90 nm) were cut with a microtome, mounted on glow-discharged carbon-coated grids, and double-stained with 2% uranyl acetate and lead citrate. Sections were imaged using a Philips Tecnai 12 BioTWIN transmission electron microscope at the Electron Microscopy Facility of the Pontificia Universidad Católica de Chile.

### India ink staining

Five microliters of *T. sanguinis*, *C. difficile*, and *B. subtilis* spores from frozen stock (5 × 10^9^ spores/mL) were diluted 1:100 in sterile Milli-Q water and mixed with an equal volume of India ink (Sigma-Aldrich, Chile), as previously described (46). Samples were mounted on 1.7% agarose pads (47) and examined by phase-contrast microscopy as described by Shuster et al. (46) for visualization of the characteristic spore halo.

### Fluorescence microscopy of sporulation stages

An aliquot of 100 μL from a 1:100 dilution of an overnight culture of *T. sanguinis* K031 in YCFA broth was spread onto YCFA agar plates and incubated anaerobically at 37 °C for 12, 24, and 48 h. After incubation, 2 mL of sterile Milli-Q water was added to each plate, and colonies were scraped and transferred to microcentrifuge tubes under anaerobic conditions. Samples were centrifuged at 3,780 × *g* for 10 min, the supernatant was removed, and the volume was adjusted to 1 mL with sterile Milli-Q water. For fluorescence staining, 50 μL of culture collected at 12, 24, or 48 h was centrifuged at 7,000 × *g* for 5 min, and the pellet was resuspended in 50 μL of staining solution containing 10 μg/mL FM 4-64 (phospholipid stain, Thermo Fisher, Chile), 9 μM MitoTracker Green (MTG, protein stain, Thermo Fisher, Chile), and 4.5 μM Hoechst (DNA stain, Thermo Fisher, Chile). Samples were incubated at room temperature for 2 min in the dark, centrifuged at 3,780 × *g* for 5 min, and the supernatant was discarded. Pellets were resuspended in 20 μL of 1× PBS, mounted on 1.7% agarose pads (47). The samples were observed on an Olympus BX53 fluorescence microscope with UPLFLN 100× oil objective (numerical aperture 1.30), and images were captured using the microscope camera for fluorescence imaging, Qimaging R6 Retiga. FM 4-64 fluorescence was captured using a fluorescence filter cube U-FGW (excitation: 530–550 nm, emission: 575 nm to infrared) with an exposure time of 600 ms. MTG fluorescence was captured using a fluorescence filter cube U-3n31001 FITC C144712 (excitation: 470–495 nm, emission: 510–550 nm) with an exposure time of 600 ms. Hoechst fluorescence was captured using a DAPI fluorescence filter cube U-3N31000v2 C144706 (excitation: 360–370 nm, emission: 420–460 nm) with an exposure time of 100 ms. Fluorescently stained morphotypes observed during sporulation were visualized and merged using ImageJ, images were organized according to canonical sporulation stages described in spore-forming bacteria (10, 12), and morphotypes of each stage were quantified and plotted using Prism 8 software (GraphPad).

### Dipicolinic acid (DPA) assay

DPA content of spore cores was quantified as previously described (48). Briefly, 5 μL of purified *T. sanguinis* spores (5 × 10^9^ spores/mL) were diluted 1:200 in 1× TBS buffer and denatured by heating at 100°C for 30 min. Samples were centrifuged at 14,000 × *g* for 5 min, and supernatants were collected (49). Aliquots of 125 μL were transferred to a 96-well plate and supplemented with 800 μM TbCl₃. The Ca-DPA concentration was determined by monitoring the fluorescence of the DPA–Tb³^+^ complex (excitation: 270 nm, emission: 545 nm) using a Synergy H1 Hybrid Multi-Mode Reader (BioTek) (48). Assay was performed in biological triplicate, each with technical triplicates.

### Spore colony formation efficiency

The colony formation efficiency of *T. sanguinis* spores was evaluated using freshly purified spores (0 days of storage) and spores stored at 4°C for 2, 4, 7, or 28 days. Aliquots of spores (1 × 10^7^ spores/mL) were plated on YCFA agar with or without prior heat activation (50°C, 60°C, or 70°C for 20 min) and incubated anaerobically at 37°C for 24 h. Spore viability was calculated as ([c.f.u./mL]/[spore particles/mL]) × 100 and expressed relative to wild-type strain.

### Spore germination

An aliquot of 5 µL of a stock of 5 × 10^9^ of purified *T. sanguinis* spores per mL was heat-activated at 60°C for 20 min and cooled at room temperature for 10 min. Heat and non-activated spores were pre-incubated at 37°C for 10 min prior to mixture with germination mixtures. All germination experiments were carried out at 37°C. Both heat- and non-heat-activated spores were incubated in 1 mL of 70:30 or BHIS, culture media typically used for *Clostridia* and *Bacillus* spore germination (50), or YCFA nutrient-rich media. OD_595_ of each sample was measured at 30, 60, 120, and 240 min using 1× PBS as optical density control. Additionally, after 4 h of incubation, aliquots were analyzed in an agarose pad under the phase-contrast microscope, and phase-bright, -gray, and -dark spores were counted (*n* = 200 spores were counted per treatment condition).

### Ortholog identification pipeline for sporulation and germination-related proteins

The predicted proteome of *T. sanguinis* K031 (JAGBKO000000000) was functionally annotated against KEGG/KO, COG, Pfam, and NCBI Conserved Domain Database. Ortholog identification proceeded in two stages. The first stage was to compile a curated data set of hallmark sporulation, germination, and spore-structure proteins from five spore-former model species *B. subtilis* strain 168 (NC_000964), *B. cereus* ATCC14579 (NZ_CP034551), *B. anthracis* Ames ancestor (NC_007530), *C. difficile* strain 630 (NC_008262), and *C. perfringens* SM101 (NC_009089) (Table S4). Each reference protein was mapped to functional profiles (KEGG Orthology, COG, Pfam, and NCBI Conserved Domain Database) (Table S5). Homology relationships were evaluated using BLASTP and HMMER (hmmsearch). Proteins were retained as candidates when they matched the same KO/COG/Pfam/CD signature as a reference or produced a significant HMM hit to the corresponding profile (default thresholds: E-value ≤1e-5 for BLASTP and hmmsearch; alignment coverage ≥50% of the reference/domain; domain architecture consistent with the reference model). All candidates were manually curated using gene-neighborhood and operon context and synteny with model organisms; final calls are reported in Table S6.

## RESULTS

### Gene and genomic evidence confirms K031 as a *T. sanguinis* representative

During the construction of our gut microbiota collection, serially diluted fecal samples from healthy donors were plated into YCFA plates, and individual colonies subjected to full-length 16S rRNA gene amplicon sequencing with universal primers 7F–1510R and identified one of the isolates as *T. sanguinis*. This isolate was whole genome sequenced to construct a high-quality hybrid genome of *T. sanguinis* K031 (3.3 Mb; 34.4% G + C) that was assembled into eight scaffolds (N50 = 2,145,658) at >99 × depth of coverage from 129.9 Mb of Illumina and 20.3 Gb of PacBio raw reads (Table S1A). The graft genome sequence was deposited at the GenBank database under the accession IDs JAGBKO000000000.

Nine 16S rRNA gene copies were identified in the *T. sanguinis* K031 genome. Clustering of these copies resolved two allelic 16S rRNA gene groups: eight identical sequences (Group 1) and a single allele with minor divergence (J3478_02830; >98% identity to Group 1) (Table S2A). Phylogenetic analyses incorporating representatives of both groups, together with reference publicly available *Turicibacter* 16S rRNA sequences and carefully curated outgroups (Table S2B), consistently placed strain K031 within the *T. sanguinis* clade (Fig. S1A). These results confirm the assignment of K031 to *T. sanguinis* species.

**Fig 1:**
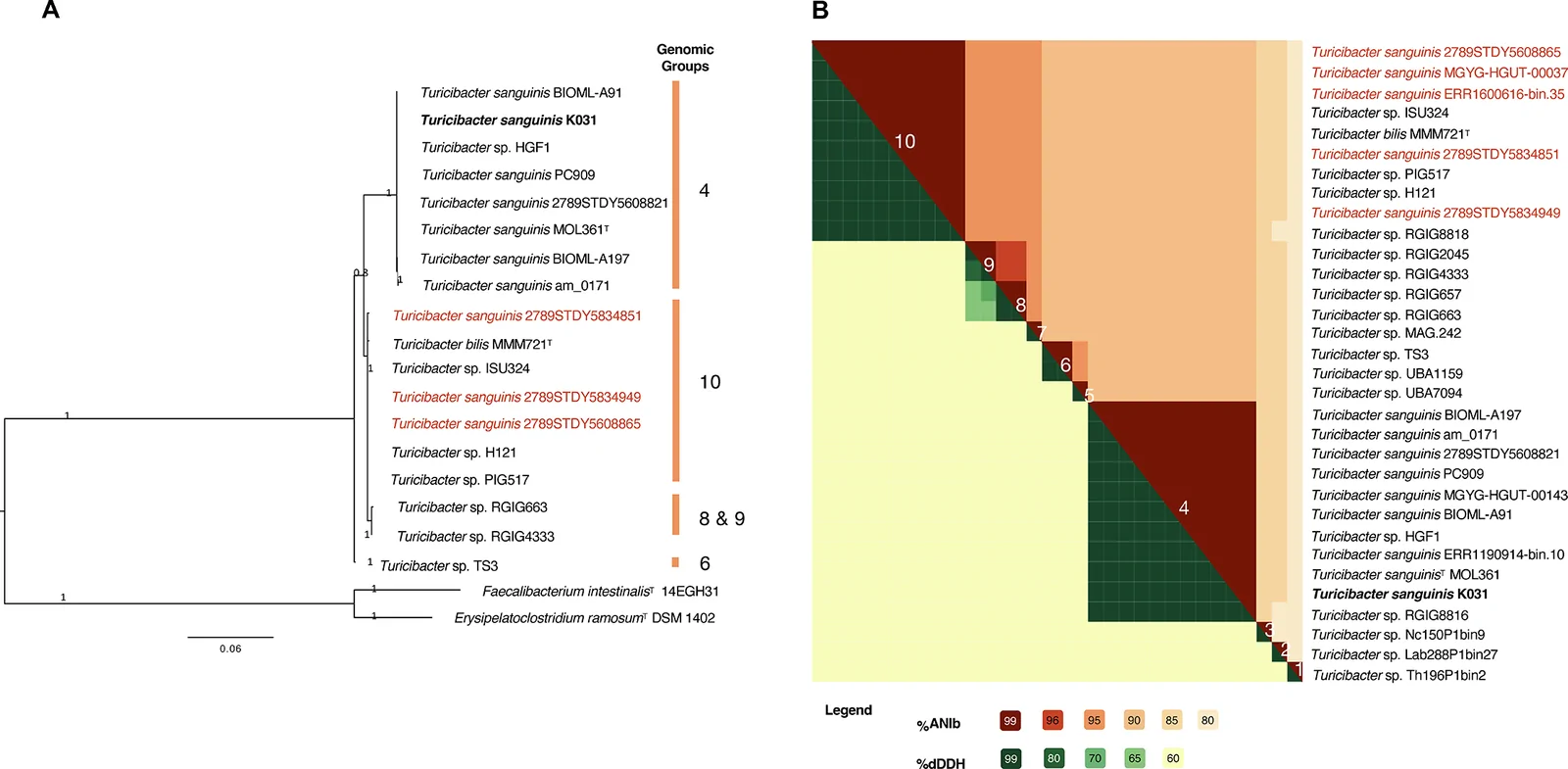
Phylogenetic and genome relatedness analysis of *T. sanguinis K031*. (**A**) MCC Bayesian phylogenetic tree with a concatenated alignment of seven ribosomal proteins present in all *Turicibacter* sp. strains and outgroups in the analysis (Table S2C). (**B**) Heatmap of Average Nucleotide Identity or ANIb (%, orange scale) and the DNA-DNA hybridization index or dDDH (%, green scale). Genomic groups in both panels are numbered similarly (from 1 to 10). Thresholds used for species delimitation are the following: ANI > 96% (same genomic species); dDDH > 70% (same genomic species). Type strains are marked with a T superscript. *Faecalibacterium intestinalis*^T^ 14EGH31 and *Erysipelatoclostridium ramosum*^T^ DSM 1402 were used as outgroups.

To generate a more comprehensive phylogeny of *T. sanguinis* and enable direct comparison of strain K031 with other sequenced strains and meta-assembled genomes tentatively assigned to the species, we retrieved a set of phylogenetically informative ribosomal proteins (RPs) (Table S2C) (51). Concatenated alignments of seven universally present RPs (RP7) were analyzed using BI, ML, and NJ methods. Although ML and NJ yielded trees with relatively unstable support (Fig. S1B and C), BI produced a robust and well-supported phylogeny (Fig. 1A). In all three trees, strain K031 consistently clustered within the *T. sanguinis* clade together with the type strain MOL361^T^ (52) (Fig. 1A and Fig. S1B and C). Unclassified *Turicibacter* strains, including one recently reassigned to *T. bilis* (53), formed a broader sister clade (Fig. 1A).

ANI and dDDH whole-genome relatedness analysis between K031 strain and publicly available *Turicibacter* spp. genome assemblies (as of February 2024, Table S1), yielded concordant results, resolving 10 genomic groups above species-level relatedness thresholds (Fig. 1B). These analyses confirmed the genomic coherence of *T. bilis* (Group 10) and *T. sanguinis* (Group 4), with strain K031 unambiguously placed within *T. sanguinis* (Group 4). Beyond these named taxa, several additional groups appear, which are represented by a single genome and lack support from the ribosomal-protein phylogeny (Fig. 1), indicating that their taxonomic status remains provisional pending additional sampling.

### *T. sanguinis* cellular morphology suggests S-layer

**Fig 2:**
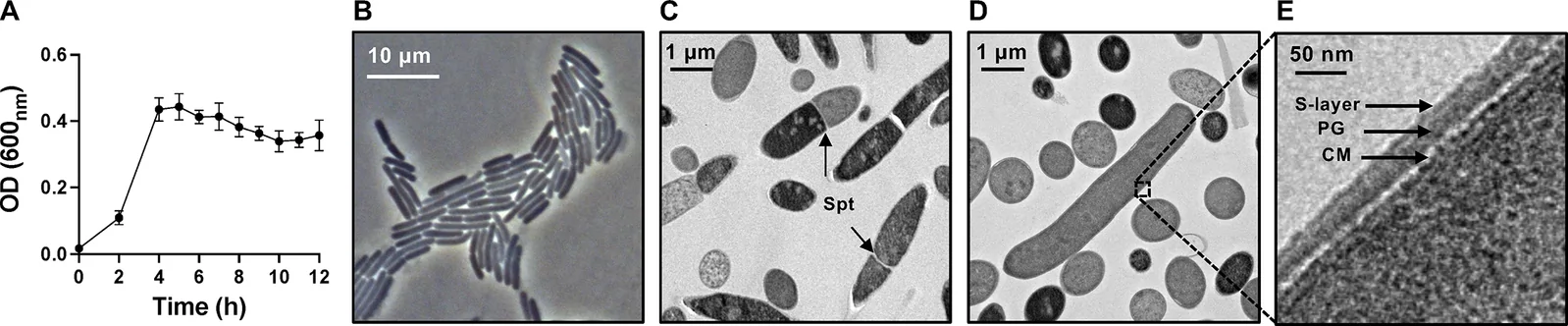
Vegetative growth of *T. sanguinis* K031 in YCFA broth**.** (**A**) Growth curve of *T. sanguinis* K031 after 2, 4, 6, 8, 10, and 12 h of incubation in YCFA broth at 37°C under anaerobic conditions. Error bars indicate the standard error of the mean (SEM). The experiment was done three times with technical triplicates. (**B**) Phase-contrast microscopy imaging of an 8 h YCFA culture of *T. sanguinis* K031. (**C**, **D**, and **E**) The figures represent transmission electron micrograph images of an 8 h YCFA culture of *T. sanguinis* K031 with a magnification of 3,400×. (**C**) Arrows denote the symmetric septum (spt) formation during cell division. Panels D and E represent vegetative cells, where (**E**) is the zoom of bacilli’s surface from (**D**). (**D**) Spherical morphologies represent a transversal cut section of *T. sanguinis* bacilli. (**E**) S-layer, peptidoglycan (PG), and cell membrane (CM) are highlighted with black arrows. Bar scales are highlighted.

To begin characterizing the basic physiology of *T. sanguinis*, we investigated its growth kinetics. An overnight culture was diluted 1:100 into pre-warmed YCFA broth and incubated anaerobically at 37°C. The exponential growth phase lasted for the first 4 h, reaching a maximum OD_600_ of 0.44, which remained stable during the subsequent 8 h of monitoring (Fig. 2A). After 16 h, however, cultures appeared to undergo lysis, consistent with the weak Hoechst staining observed (Fig. S2A). Planktonic cells from 8 h cultures were examined by phase-contrast microscopy (Fig. 2B) and transmission electron microscopy (Fig. 2C through E). Phase-contrast imaging confirmed a bacillary morphology (Fig. 2B), while TEM revealed that cell division is preceded by symmetric binary fission (Fig. 2C). TEM also showed numerous spherical and rod-shaped profiles (Fig. 2C and D), which likely reflect cross-sectional orientations of cells in thin resin sections. At higher resolution, TEM further suggests that the cell wall of *T. sanguinis* has an outer S-layer-like structure surrounding the peptidoglycan layer and underlying cytoplasmic membrane (Fig. 2E).

### *T. sanguinis* forms phase-bright spores

**Fig 3:**
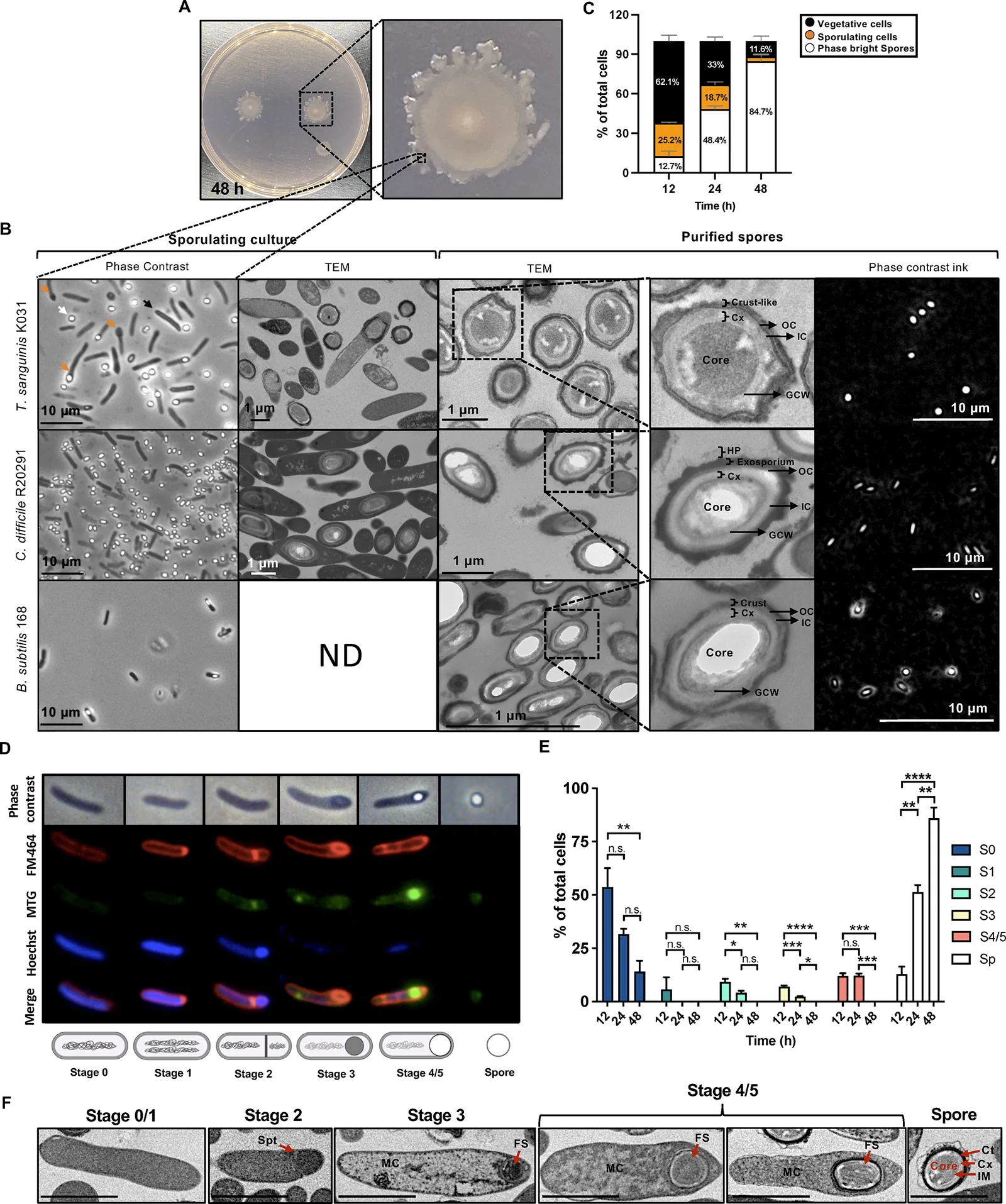
Ultrastructural and physiological characterization of *T. sanguinis* K031 sporulation. (**A**) Representative *T. sanguinis* K031 48 h colony in YCFA agar plate. (**B**) Visualization of sporulating cultures and purified spores of *T. sanguinis*, *C. difficile,* and *B. subtilis* by phase-contrast microscopy and transmission electron microscopy. First, two left panels represent phase-contrast microscopy and TEM images of *T. sanguinis* K031, *C. difficile* R20291, and *B. subtilis* strain 168 sporulated cultures after 48 h of anaerobic incubation at 37°C on YCFA, 70:30 or LB plates for each strain, respectively. Black, orange, and white arrows denote vegetative cell, sporulating cell, and free spore respectively. Subsequent two panels correspond to TEM images from purified spores, and individual zoomed spore of *T. sanguinis* K031, *C. difficile* R20291 and *B. subtilis* strain 168 harvested. In the zoom of individual spores are pointed out each structural layer. Core, germ cell wall (GCW), cortex (Cx), inner coat (IC), outer coat (OC), or electron-dense OC (ED-OC), exosporium, hair-like projections (HP), and crust or crust-like. Panels further to the right evaluate the presence of crust in purified spores of *T. sanguinis*, *C. difficile* R20291, and *B. subtilis* using India ink (presence of polysaccharides). *B. subtilis* strain 168 and *C. difficile* strain R20291 spores were positive and negative controls for halo formation around the spore, respectively. TEM images of sporulating culture of *T. sanguinis* K031 and *C. difficile* R20291 were taken at a magnification of 3,400× and 11,500×, respectively. TEM images of purified spores of *T. sanguinis* K031, *C. difficile* R20291, and *B. subtilis* strain 168 were taken at a magnification of 6,700×, 20,500×, and 60,000×, respectively. Bar scales are highlighted. (**C**) Phase-contrast microscopic quantification of sporulation efficiency of *T. sanguinis* K031 during 12, 24, and 48 h of incubation at 37°C under anaerobic conditions. Average from three independent experiments and error bars indicate the standard error of the mean (SEM). (**D**) Sporulation developmental stages in *T. sanguinis*. Representative micrographic images of 12, 24, and 48 h of YCFA agar plate sporulating cultures of *T. sanguinis* were stained with Hoechst (DNA), FM4-64 (membrane-impermeable dye), and MTG (membrane-permeable dye). Schematic development and examined by phase-contrast and fluorescence microscopy. Schematic representation of the developmental stages is depicted below selected micrographs. (**E**) Frequency of developmental stages at various time points in YCFA agar plates. Error bars indicate the standard error of the mean (SEM). The graph represents three independent experiments. Statistical analysis was performed using one-way ANOVA followed by Tukey’s multiple comparison test, ns: no significance, *P* < 0.5: *P < 0.1, **P < 0.01, ***P < 0.001, ****P < 0.0001. (**F**) Transmission of electronic micrographs at a magnification of 3,400× of 48 h sporulation culture of *T. sanguinis* K031 in YCFA plates. Representative images of the different developmental stages are shown. MC: mother cell, FS: forespore, Spt: septum, Ct: coat, Cx: cortex, IM: inner membrane. Scale bars represent 2 µm.

Among the different colony morphologies, *T. sanguinis* formed flat colonies with a white-irregular-shiny viscous texture, and a wavy margin, with a bubble in the center of the colony (Fig. 3A). A notable observation when culturing *T. sanguinis* on YCFA agar plates was the appearance of phase-bright structures resembling dormant spores under phase-contrast microscopy (Fig. 3B and Fig. S2B), which contrasted with the absence of sporulating cells in YCFA broth cultures (Fig. 2B and Fig. S2A). To investigate the sporulation dynamics of *T. sanguinis*, overnight YCFA broth cultures were diluted 1:100, plated onto YCFA agar, and incubated anaerobically for 12, 24, and 48 h at 37 °C. Phase-contrast microscopy revealed the appearance of sporulating cells (25%) and phase-bright free spores (~13%) as early as 12 h of incubation, and after 48 h, phase-bright free spores reached ~85% of total (Fig. 3C).

To further characterize sporulation, we compared *T. sanguinis* sporulating cultures with those of *C. difficile* strain R20291 and *B. subtilis* 168 using phase-contrast and TEM (Fig. 3B). Phase-contrast microscopy and electron micrographs demonstrate that *T. sanguinis* forms endospores similarly to *C. difficile* and *B. subtilis* (Fig. 3B). TEM analysis showed that *T. sanguinis* spores possess the same general structural organization as those of *B. subtilis* and *C. difficile*: a spore core surrounded by an inner membrane, a germ cell wall, a thick cortex peptidoglycan layer, inner and outer coat layers, and an outermost layer resembling the *B. subtilis* crust rather than the exosporium observed in *C. difficile* spores (Fig. 3B).

In *B. subtilis* and its close relatives (referred to as the *B. subtilis* group), the outermost crust or exosporium layer is adorned with polysaccharides, where sugars like rhamnose are common in these species (54). A traditional method to identify the presence of polysaccharides in the spore surface is using India ink, which is unable to penetrate through the polysaccharide layer, thus producing a white halo around the spores (46). Therefore, since electron micrographs suggest that the outermost structure of the *T. sanguinis* spores is similar to the crust observed in *B. subtilis*, we assayed *T. sanguinis* spores with India ink to test whether the *T. sanguinis* spore’s outermost layer would also contain polysaccharides as those of the *B. subtilis* crust. However, as we can observe in Fig. 3C, unlike the halo observed in *B. subtilis* spores, neither *T. sanguinis* nor *C. difficile* spores exhibited a halo surrounding the spore (Fig. 3B). These results suggest that although the morphology of the crust of *T. sanguinis* spores is similar to that of *B. subtilis,* it might not be adorned with polysaccharides. Despite this slight biochemical difference, given the ultrastructural similarities of the outermost layer of *T. sanguinis* and *B. subtilis* spores, the outermost layer of *T. sanguinis* spore will be named as “Crust-like” outermost layer.

### Developmental stages of sporulation in *T. sanguinis* follow canonical models

Given that *T. sanguinis* forms similar spores to those from model spore-formers *B. subtilis* and *C. difficile,* as was mentioned before (Fig. 3C), we next asked whether its sporulation process also followed the canonical sequence described in Bacilli and Clostridia. To address this, *T. sanguinis* was grown under sporulating conditions on YCFA plates, the medium in which spore formation had previously been observed. Because phase-bright spores were already evident after 12 h of incubation, we monitored sporulation at 12, 24, and 48 h under anaerobic conditions at 37 °C using transmission electron microscopy and fluorescence microscopy with Hoechst, MTG, and FM 4-64 staining to visualize DNA, proteins, and membranes, respectively (Fig. 3D). Based on these analyses, we identified five sporulation stages. Stage 0 (S0) represented vegetative cells, while stage 1 (S1) showed increased Hoechst staining consistent with chromosome duplication. Stage 2 (S2) was characterized by septum formation at one pole of the cell, stained with FM 4-64 and MTG, indicating its lipid and protein composition. Stage 3 (S3) corresponded to an engulfed forespore, evidenced by FM 4-64 staining of the surrounding membrane. Stage 4/5 (S4/5) were presented by a phase-gray and phase-bright endospore within the mother cell, respectively, with intense MTG (Fig. 3D and F). To quantify the distribution of morphotypes over time, we scored cells at each stage after 12, 24, and 48 h of incubation (Fig. 3E). While the relative abundance of S0 decreased after 48 h of sporulation, stage S1 remained constant, whereas stages S2, S3, and S4/5 decreased similarly to S0 after 48 h of sporulation (Fig. 3E). In the case of free spores, these increased up to ~80% of the population after 48 h (Fig. 3E). These results demonstrate that *T. sanguinis* sporulation is asynchronous and progresses from vegetative growth toward spore formation over time in YCFA plates, following the canonical stages of sporulation (Stages S1–S5).

### Architecture of *T. sanguinis* spores

**Fig 4:**
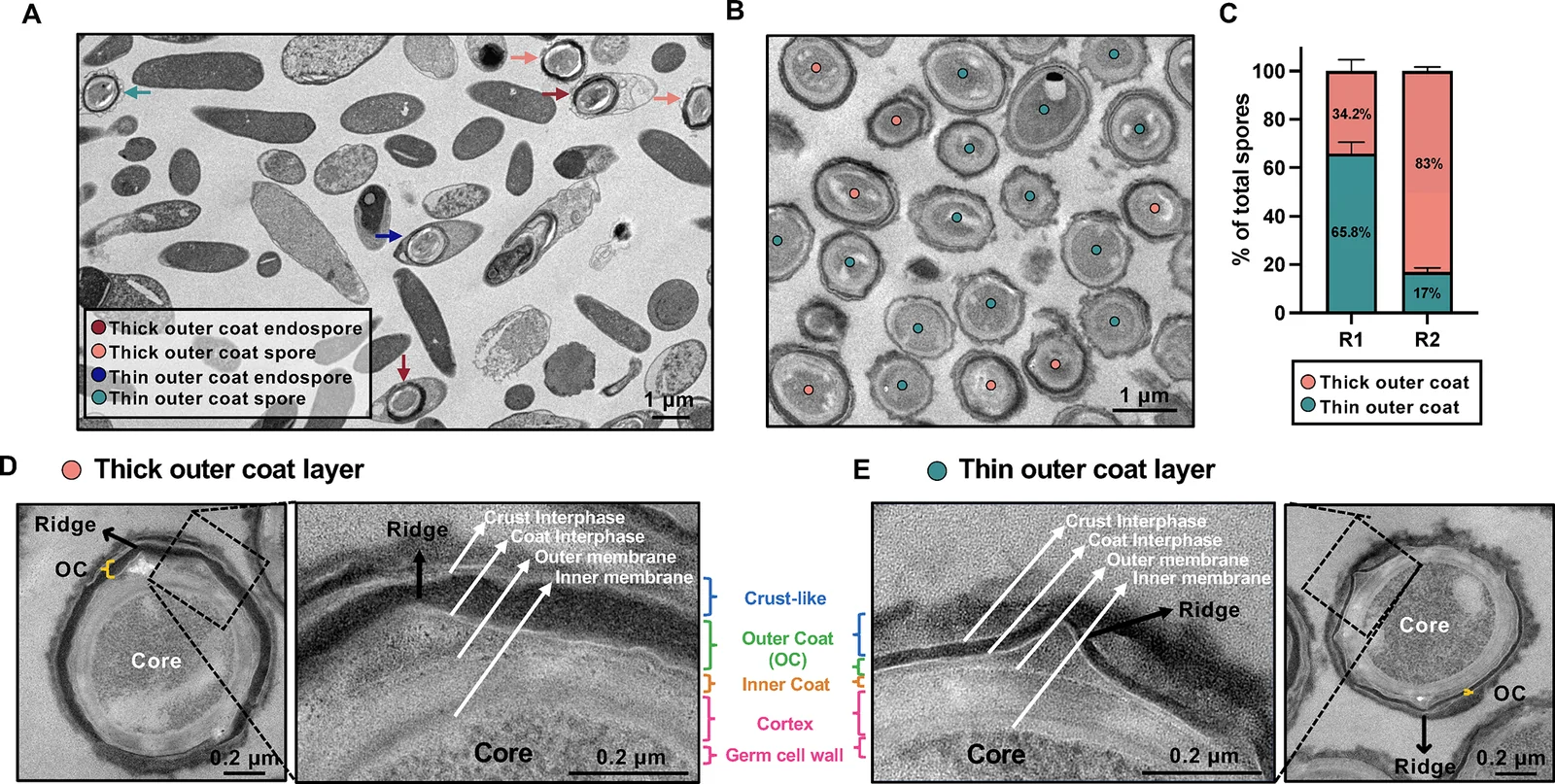
Transmission electron microscopy reveals thick and thin *T. sanguinis* K031 spore morphotypes**.** (**A**) Electron micrographs of *T. sanguinis* K031 sporulated culture, grown for 48 h in YCFA plate, with a magnification of 3,400×. Thin and thick ED-OC endospores and spores are highlighted. (**B**) Representative TEM image of *T. sanguinis* K031 purified spores with a magnification of 5,300×. *T. sanguinis* spores with a thick (coral circles) or thin (green circles) electron-dense outer coat (ED-OC) layer are highlighted. (**C**) Quantification of thick and thin outer coat spores with respect to the total spore population. A total of 335 spores were visualized from two biological replicates of purified spores (R1: *n* = 221; R2: *n* = 114). Error bars indicate the standard error of the mean (SEM). Bars represent percentage of thick (coral) and thin (green) outer coat spores in the population. (**D and E**) Zoom of thick (Panel D) and thin (Panel E) outer coat spores. The distinctive spore layers, membranes, and interphases are shown: ridge, crust-like (Cl), crust interphase, coat interphase, outer coat (OC), inner coat (IC), outer membrane, cortex (Cx), germ cell wall (GCW), and inner membrane and core.

TEM analysis of sporulating *T. sanguinis* cultures revealed that endospores within mother cell sporangia displayed either a thick or thin electron-dense surface layer (Fig. 4A), suggesting the presence of two distinct spore morphotypes, as previously described in *C. difficile* (44). Interestingly, upon measuring purified *T. sanguinis* spores by transmission electron micrographs, we observed that the entire spore population could be divided into thin or thick outer surface spores (Fig. 4B and Fig. S3). Quantification of two independently prepared batches of *T. sanguinis* spores demonstrated that depending on the spore preparation, 34%–83% of the spores had a thick electron-dense outer coat layer (Fig. 4C), denoting potential phase variations in this trait.

At higher magnification (36,000×), thick-type spores exhibited a prominent electron-dense layer (Fig. 4D), whereas this layer was much thinner in thin-type spores (Fig. 4E). Despite this difference, both morphotypes shared a similar internal organization: a spore core bounded by an inner membrane, surrounded by a cortex-like peptidoglycan layer, an outer membrane, and multiple concentric coat layers including the inner coat, the electron-dense outer coat, and an intermediate coat layer (Fig. 4D and E). Notably, the first layer external to the outer membrane lacked the lamellae-like features characteristic of *B. subtilis* and *C. difficile* spores (13, 44, 55, 56).

A striking feature in both morphotypes was the presence of surface ridges originating from the layer beneath the electron-dense outer coat layer (Fig. 4D and E). Ridge counts followed an average of nine ridges per spore. Surrounding the electron-dense outer coat was a diffuse outermost layer resembling the *B. subtilis* crust, separated by a defined crust interphase in both type-spores (Fig. 4D and E). Altogether, these observations indicate that *T. sanguinis* spores exhibit a hybrid architecture, sharing structural features of both *B. subtilis* and *C. difficile* spores, and, as in *C. difficile*, forming two distinct morphotypes characterized by thin or thick electron-dense outer layers.

### Analysis of *T. sanguinis* spore architecture reveals thick/thin spores and an outer crust

**Fig 5:**
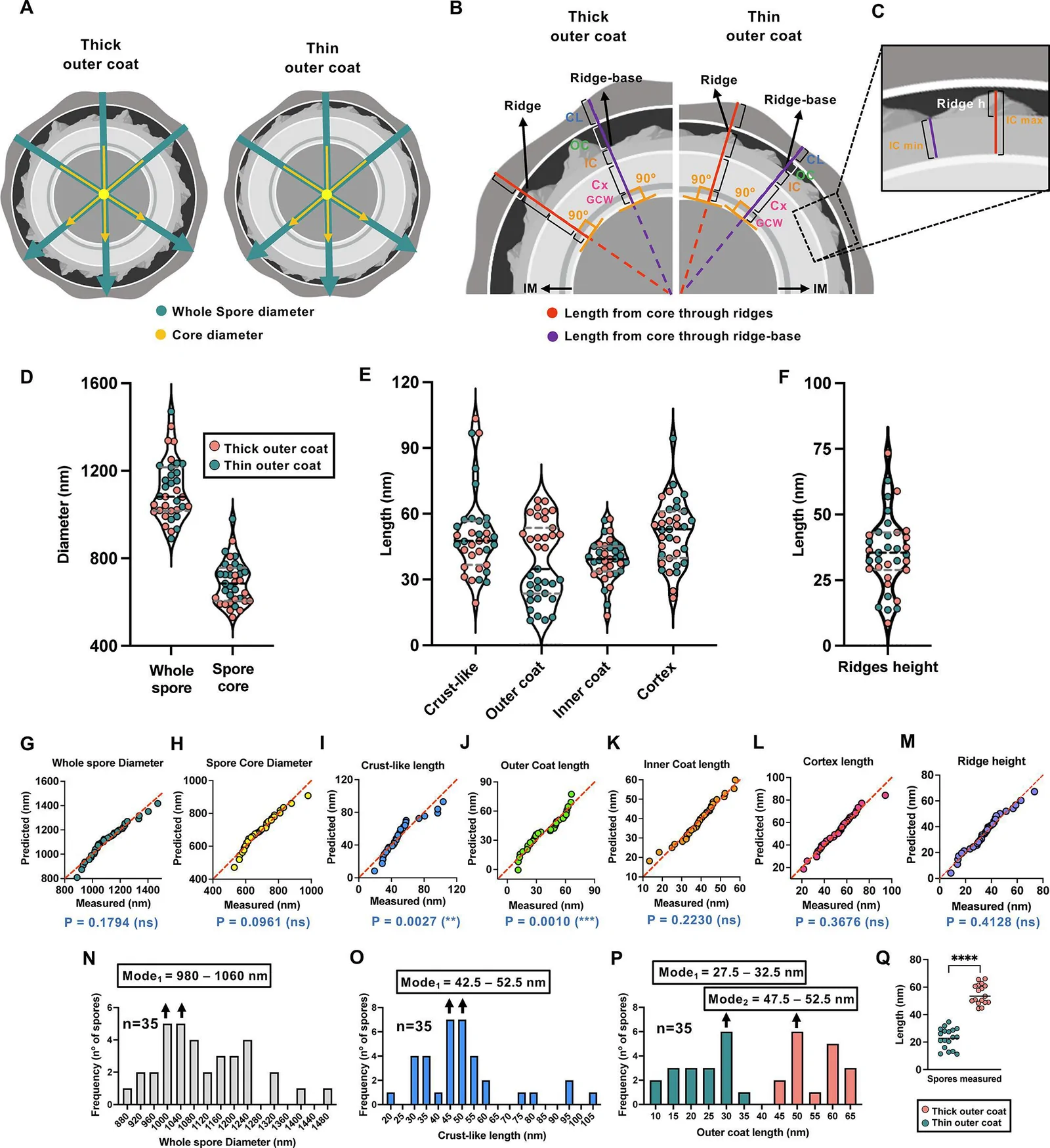
*T. sanguinis* K031 spore quantitative analysis of ultrastructural layers. (**A**) Schematic representation of measurement of whole-spore length quantification in both thick and thin outer coat morphotypes in *T. sanguinis*. First, three concentric vectors are drawn in each spore to measure whole spore (green arrows) and spore core (yellow arrows) diameters. Measurements for each spore and spore core diameter represent the average of three measurements per spore. (**B**) Model representation of thin and thick outer coat spores’ structural layers length measurement. First, six lines are drawn to measure spores cortex (Cx), inner coat (IC), outer coat (OC), and crust-like (Cl) layers. Three lines crossing from the top of the ridge (red line), and three lines crossing the bottom of the ridges (purple line). Measurements for each spore represent the average of six measurements per spore. (**C**) Model representation of ridge height, where height for each ridge was measured as the difference between the lengths between the maximum (red line) and minimum (purple line) length of the IC. (**D–F**) Layer-length quantification of a total of 35 individual *T. sanguinis* spores from two biological replicates (R1: *n* = 22; R2: *n* = 13). The length of whole spore and spore core (Panel D), Cl, OC, IC, and cortex (Cx) (Panel E), and of the ridge height (Panel F) was quantified. In D–F, thick and thin spores are represented by coral and green circles, respectively. Black- and gray-dashed lines represent median and quartiles of spores measured, respectively. (**G–M**) QQ plots of the normal distribution of the length of the diameters of whole spore (**G**) and spore core (**H**) diameters, the length of crust-like layer (**I**), outer coat (**J**), inner coat (**K**), cortex (**L**), and height of ridge (**M**). Plots were tested for normal distribution by D'Agostino & Pearson test; *P* < 0.05 is indicative of departure from normality. Each circle symbol represents the average length of one spore. (**N–P**) Histograms of frequency distribution of the length of whole spore (**N**), crust-like (**O**), and outer coat (P) were plotted using Prism 8 software and modes for skewed (**O**) and bimodal distributions (**P**) were highlighted. Green and coral bars from panel P represent thin and thick outer coat spores, respectively. (**Q**) Graph representing thin (green circles) and thick (coral circles) outer coat *T. sanguinis* spores’ populations. Statistical analysis was performed by two-tailed paired Student’s *t*-test, ****P < 0.0001.

Ultrastructural observations revealed that *T. sanguinis* produces two distinct spore morphotypes characterized by either a thin or thick electron-dense outer coat. To determine whether these differences followed a structured pattern within the population, we performed a quantitative morphometric analysis of spore ultrastructure (Fig. 5A through C). For whole-spore diameter and spore core size measurements, each spore was divided into three symmetrical axes through the center, and diameters were averaged (Fig. 5A). Measurements were performed on both thin and thick electron-dense outer coat spores, which were color-coded for comparative analysis (green, thin; coral, thick) (Fig. 5D). To assess the thickness of structural layers beyond the core, including the cortex, inner coat, electron-dense outer coat, and crust-like layers, measurements were made along lines drawn through ridges and inter-ridge regions, averaging six per spore (Fig. 5C). Ridge height was calculated as the difference between maximum and minimum inner coat thickness.

Quantitative analysis showed that *T. sanguinis* spores had an average whole-spore diameter of ~ 1.1 µm (range: 0.9–1.5 μm) and a spore core diameter of ~ 0.7 µm (0.5–0.9 μm) (Fig. 5D). The crust-like averaged 51 nm (19–103 nm), the electron-dense outer coat 38 nm (11–66 nm), the inner coat 39 nm (13–58 nm), and the cortex 51 nm (22–94 nm) (Fig. 5E). Ridge height averaged 36 nm (9–73 nm) (Fig. 5F). These values are comparable to those reported for *C. perfringens* and *C. difficile* spores (44, 57). Inspection of thickness distributions revealed high heterogeneity across layers. Normality testing (QQ plots) showed that whole-spore diameter, spore core diameter, inner coat thickness, cortex thickness, and ridge height followed normal distributions (Fig. 5G and H and K through M). In contrast, crust-like and electron-dense outer coat thicknesses deviated from normality (Fig. 5I and J). The whole-spore diameter histogram confirmed a clear normal distribution (Fig. 5N), whereas the crust-like length histogram was skewed, with a mode between 42.5 and 52.5 nm (Fig. 5O), which was not observed in the other layers (Fig. S4A through D). Strikingly, the thickness of the electron-dense outer coat layer exhibited a bimodal distribution with two distinct modes (27.5–32.5 nm and 47.5–52.5 nm), suggesting two subpopulations of spores (Fig. 5P). When spores were classified by morphology, thin and thick electron-dense outer coat layer spores each followed normal distributions (Fig. S4E and F). Comparative analysis confirmed that thin electron-dense outer coat layer spores had a significantly reduced thickness (mean = 22.8 nm) compared to thick electron-dense outer coat layer spores (mean = 54.9 nm; *P* < 0.0001) (Fig. 5Q). Importantly, electron-dense outer coat layer thickness did not correlate with whole-spore diameter or other layer measurements (Fig. S5), suggesting that assembly of the electron-dense outer coat is governed by a distinct regulatory mechanism, independent of overall spore size.

### *T. sanguinis* spores germinate in the presence of nutrients

**Fig 6:**
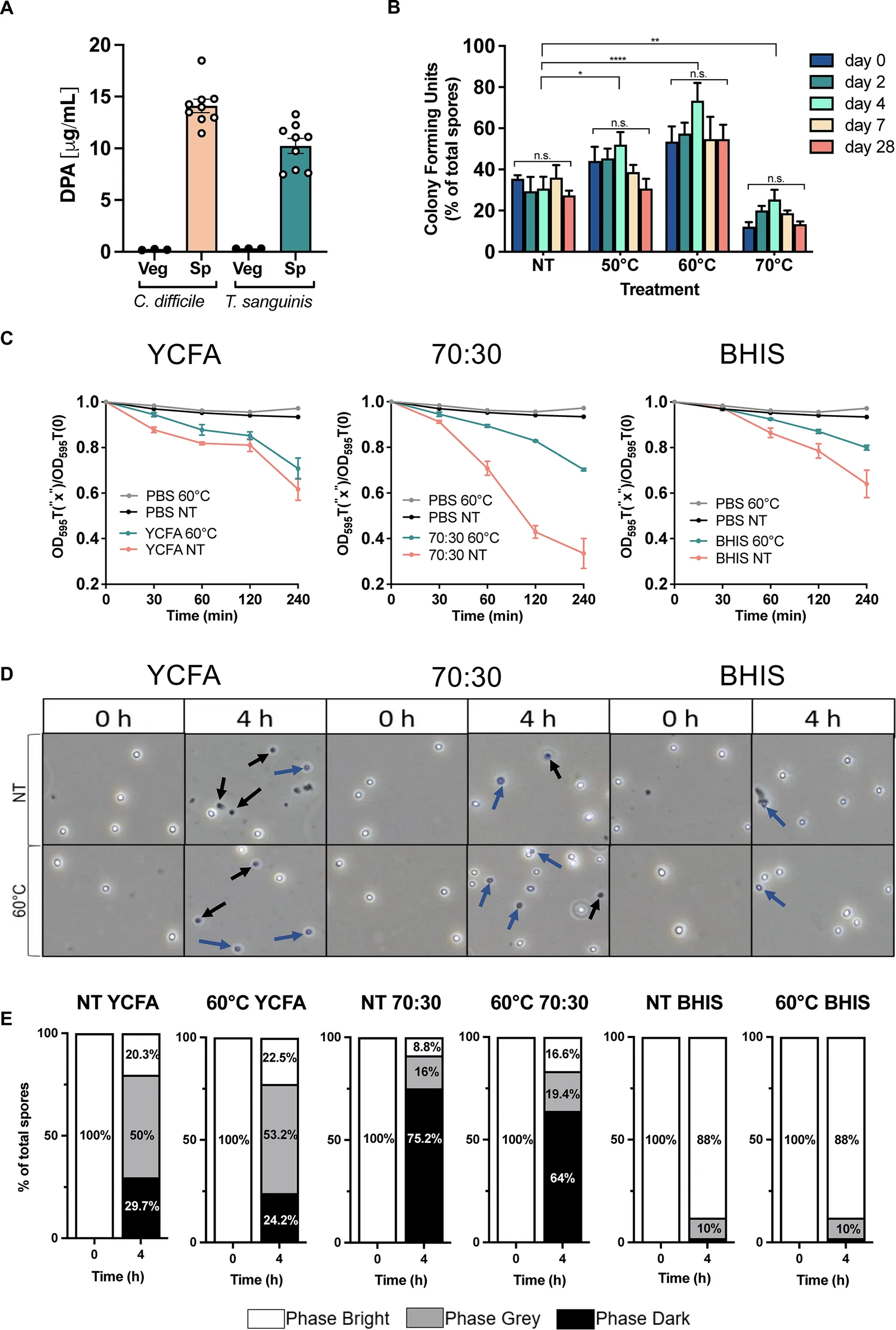
DPA content and spore germination of *T. sanguinis* spores**.** (**A**) Dipicolinic acid (DPA) content was quantified from vegetative cells and spores of *T. sanguinis* and *C. difficile*. (**B**) Colony-forming units expressed were quantified by plating untreated or heat-activated (50°C, 60°C, or 70°C) spores (10^7^ spores/mL) onto YCFA agar plates and expressing values relative to total quantified spores. (**C, D, and E**) Spore germination of *T. sanguinis* K031 on YCFA, 70:30, and BHIS culture media. *T. sanguinis* spores were heat-activated at 60°C and compared against non-activated spores (NT). Spore germination was measured by optical density (**C**) and phase-contrast microscopy after 4 h (**D**). (**E**) Quantification of germination by phase-contrast microscopy in panel D. A minimum of 200 spores were quantified per condition. All experiments were done three independent times in three technical replicates. Error bars represent the standard error of the mean. *P < 0.05; **P < 0.01; ****P < 0.0001. Black arrows: phase dark spores, blue arrows: phase-gray spores.

DPA is a hallmark of bacterial spore cores that contributes to DNA protection through dehydration (56). Therefore, to assess whether *T. sanguinis* spores also contained DPA, purified spores were heat-treated, and released DPA was quantified fluorometrically via TbCl₃-complexation assay. *T. sanguinis* spores contained ~11 µg/mL DPA, comparable to the ~14 µg/ml measured in *C. difficile* R20291 spores used as a positive control (Fig. 6A) (58), indicating that *T. sanguinis* spores share a canonical marker of bacterial spores.

Heat activation of bacterial spores synchronizes them to germinate homogeneously and increases the spore colony-forming efficiency (59). However, spores also age and lose viability through extended storage (45). Therefore, we examined how spore age and heat activation affected spore colony viability. For this, spores stored at various time points up to 28 days at 4°C were treated with several heat activation temperatures. We observed that from all heat activation conditions, 30 min at 60°C led to the highest colony-forming efficiency of up to ~70% (Fig. 6B). Higher temperature (e.g., 70°C) led to a decrease in spore colony-forming efficiency (Fig. 6B). By contrast, spore age had no effect on spore colony-forming efficiency (Fig. 6B). Notably, untreated spores had a spore colony-forming efficiency of ~35%, indicating that while heat activation might be useful *in vitro*, under physiologically relevant conditions, heat activation of *T. sanguinis* spores is not required.

The ability of spores to return to vegetative cells is essential for their biological function and requires specific germinant(s) to trigger the process (59). We assessed whether *T. sanguinis* would germinate in commonly used nutrient-rich broth such as YCFA, 70:30 (typically used to sporulate *C. difficile*), or BHIS (for growth of *C. difficile*) (60, 61). Hence, heat-activated and untreated spores were incubated in YCFA, 70:30, and BHIS broth, and OD_600_ was measured over 4 h (Fig. 6C). Unexpectedly, non-heat-treated spores showed greater OD decline than heat-treated spores, particularly in 70:30 medium, which exhibited an OD_600_ decrease of ~70% (Fig. 6C). Phase-contrast microscopy revealed three phenotypes (phase-bright, gray, and dark spores), (Fig. 6D and E). In YCFA, after incubation of non-treated spores, ~50% reached phase gray, and only ~30% became phase dark. In the presence of heat activation, ~53 and ~24% of the spores became phase gray and dark, respectively (Fig. 6E). In contrast, incubation of non-treated spores in 70:30 medium yielded 16% and 75% of phase gray and dark spores, respectively, which, with heat activation reached to ~19% and ~64%, respectively (Fig. 6E). Moreover, incubation of *T. sanguinis* spores in BHIS, regardless of the presence or absence of heat activation, led to only ~10 and ~2% of the spores changing to phase gray and dark, respectively. (Fig. 6E). Altogether, these results indicate that the kinetics of germination of *T. sanguinis* spores, unlike model organisms, do not require heat activation and that some nutrient-rich broths are sufficient to trigger germination of *T. sanguinis* spores.

### Sporulation pathway in *T. sanguinis*

**Fig 7:**
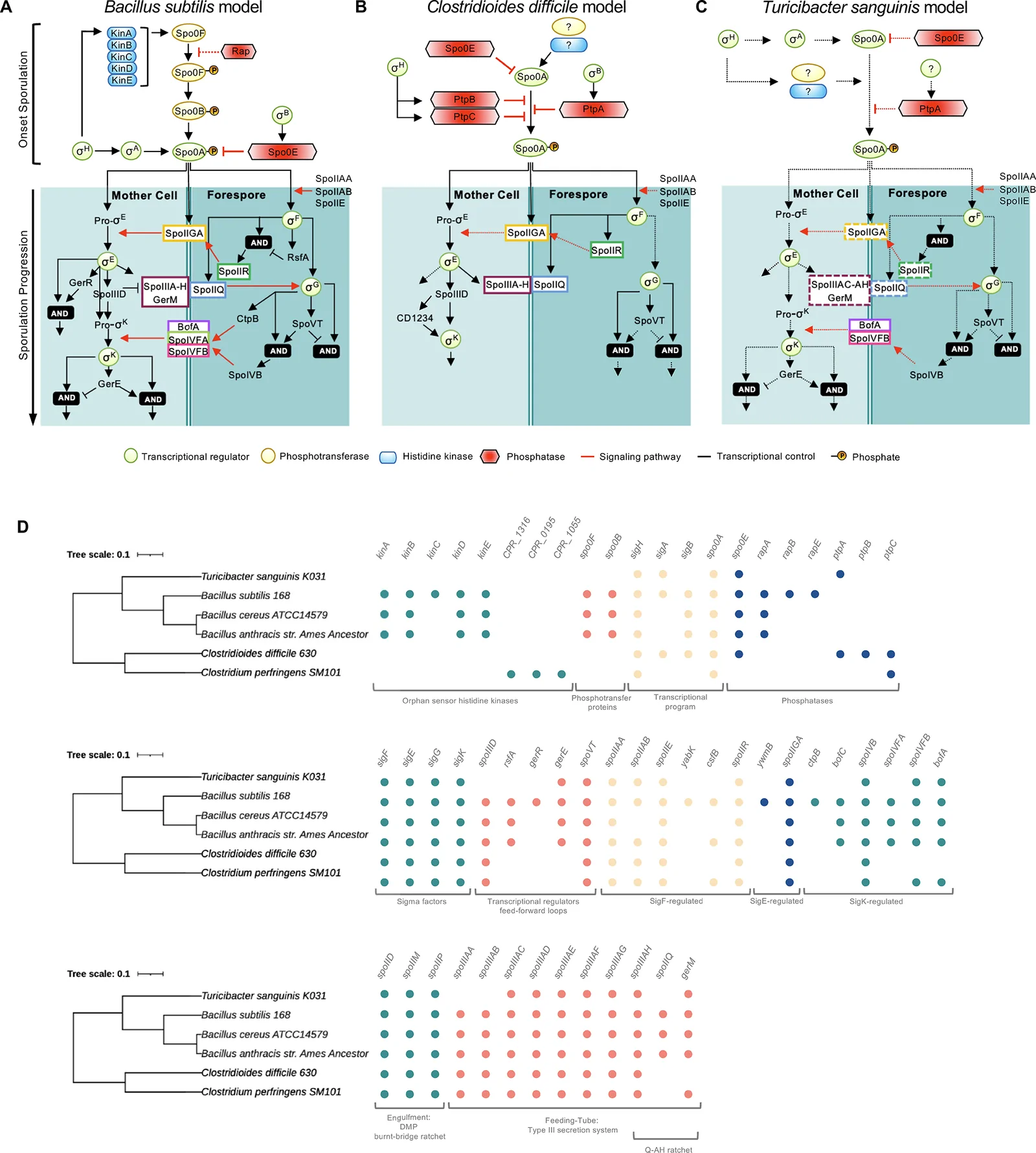
Putative sporulation pathway in *T. sanguinis***.** Schematic transcriptional regulation of sporulation in *B. subtilis* (**A**) and *C. difficile* (**B**) and suggested model of *T. sanguinis* (**C**), adapted from Shen et al. 2019 (65). Temporal progression of sporulation in the three species is shown from top to bottom. Transcriptional regulators, phosphotransferases, histidine kinases, and phosphatases from the onset of sporulation are enclosed in green, yellow, light blue, and red boxes, respectively. As described in symbology, black arrows represent transcriptional control of gene expression, red arrows indicate signaling pathways, and dashed lines indicate that the regulatory relationship remains unknown. Proteins enclosed in white boxes with colored outlines directly participate in signaling between the mother cell and forespore. (**D**) Phylogenetic tree was made with 16S ribosomal RNA gene sequence (16S rRNA) from *T. sanguinis* K031, Bacilli (*B. subtilis* strain 168, *B. cereus* ATCC14579, *B. anthracis* Ames Ancestor), and Clostridia (*C. difficile* strain 630 and *C. perfringens* SM101), using ClustalW (https://www.genome.jp/tools-bin/clustalw) for their alignment. The tree was constructed using FastTree v2.1.8 with default parameters and then graphically visualized with the web tool Interactive Tree Of Life V3 (http://itol.embl.de). *B. subtilis* strain 168, *B. cereus* ATCC14579, *B. anthracis* Ames Ancestor, *C. difficile* strain 630, and *C. perfringens* SM101 genomes were downloaded from the NCBI genome database with accession number NC_000964, NZ_CP034551, NC_007530, NC_008262, and NC_009089, respectively. Phylogenetic profile shows the conservation of gene presence for relevant functional categories.

To gain insight into the sporulation pathway, we utilized a pipeline for sporulation-related orthologues discovery in *T. sanguinis* (Fig. S6). Comparative genomic analysis revealed the absence of orthologs of the *Bacillus*-type phosphorelay components, the phosphotransfer proteins (e.g., Spo0F and Spo0B), and orphan histidine kinases (e.g., KinA–E) (Fig. 7A and Table S6). However, key regulators of sporulation onset were identified, including *sigH* (J3478_09480) and *spo0A* (J3478_15840), along with three *sigA* paralogs (J3478_13700, J3478_14895, and J3478_09755), the first of which corresponds to the *B. subtilis sigA* ortholog (Fig. 7C and D and Fig. S7 and Table S6). The *spo0E* (J3478_13625) and *ptpA*-like phosphatase (J3478_05720) orthologs, known to inhibit *spo0A* activity in *C. difficile* (62–64), were also identified (Fig. 7C and D and Table S6), suggesting that *T. sanguinis* is also subjected to similar sporulation repression as observed in these species. Altogether, these genomic features indicate that while *T. sanguinis* conserves the central regulatory logic of Spo0A-mediated sporulation, the molecular activation may be unique to this lineage.

Once Spo0A is phosphorylated, sporulation proceeds when the four conserved sporulation-specific sigma factors, σ^F^, σ^E^, σ^G^, and σ^K^ (Fig. 7A), are activated, driving compartment-specific transcription in the forespore and mother cell (66–68). Genomic analysis identified orthologs of all four sigma factors in *T. sanguinis*, σ^F^ (J3478_01570), σ^E^ (J3478_00215), σ^G^ (J3478_00210), and σ^K^ (J3478_06915) (Fig. 7C and D and Fig. S8 through S10 and Table S6). Consistent with σ^F^ regulation, the anti-sigma factors SpoIIAA (J3478_01580) and SpoIIAB (J3478_01575) were also detected (Fig. 7C and D and Fig. S11A and Table S6). Notably, unlike *B. subtilis* and *C. difficile* where *sigK* is interrupted by a prophage that splices during later stages of sporulation, in *T. sanguinis*, *sigK* is present as an intact ORF (Fig. S10). The presence of these regulatory elements aligns with the sporulation stages observed by fluorescence and electron microscopy (Fig. 3D and E) and indicates that *T. sanguinis* encodes the canonical sigma-factor cascade associated with early sporulation.

The activation of sporulation-specific sigma factors requires cross-compartment signaling between the forespore and mother cell (Fig. 7A) (65). Genomic analysis of *T. sanguinis* revealed the orthologs of several proteins involved in these signaling pathways. Components of the forespore-to-mother cell signaling axis were represented by *spoIIGA* (J3478_00220), which in Bacilli and Clostridia model organisms, is activated by σ^F^-dependent SpoIIR (J3478_04790) to trigger σ^E^ maturation (Fig. 7C and D and Fig. S9 and Table S6) (69–71). Likewise, orthologs of the σ^E^-dependent mother cell-to-forespore signaling system, including *spoIIQ* (J3478_04710), *spoIIIAC-AH* (J3478_07170–07145), and *gerM* (J3478_13875) (72–75), were identified (Fig. 7C and D and Fig. S12 and Table S6). In contrast, genes encoding the mother cell regulators controlling σ^K^ activation in *B. subtilis* but absent in *C. difficile*, while SpoIVFA was not detected, BofA (J3478_05245) and SpoIVFB (J3478_02455) were present in *T. sanguinis* genome (Fig. 7A and B and Fig. S13B and C and Table S6). These findings support the presence of conserved machinery associated with activation of the late forespore transcriptional program, including σ^G^.

After asymmetric division, sporulation proceeds with engulfment of the forespore by the mother cell (65). Genomic analysis of *T. sanguinis* K031 revealed homologs of the canonical engulfment machinery (76–78), *spoIID* (J3478_04715), *spoIIM* (J3478_15865), and *spoIIP* (J3478_07105) (Fig. 7D and Fig. S14 Table S6), indicating conservation of the septal peptidoglycan degradation system involved in this morphogenetic step.

The transcriptional program governing sporulation is modulated by σ-factor-associated feed-forward loops (79). In *T. sanguinis*, orthologs of the late-stage regulators SpoVT (σ^G^-dependent; J3478_08120) and GerE (σ^K^-dependent; J3478_13865) were identified (Fig. 7C and D and Fig. S15 and Table S6), whereas orthologs of early feed-forward regulators associated with σ^F^ and σ^E^ were not detected. These findings suggest that *T. sanguinis* retains regulatory fine-tuning during late forespore and mother-cell stages of development.

### Genomic analysis of spore surface layer homologs

**Fig 8:**
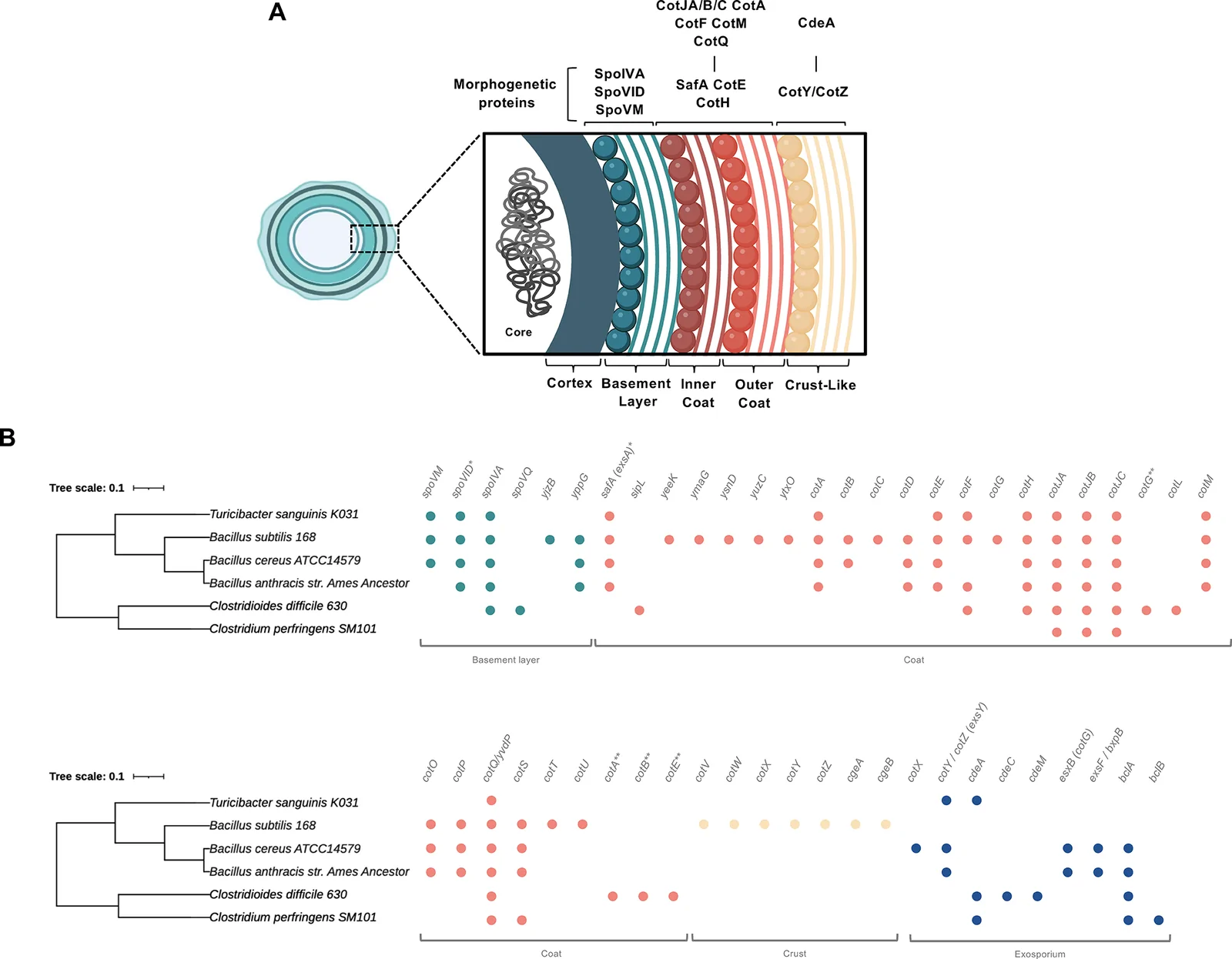
Putative spore surface layers of *T. sanguinis* spores. (**A**) Schematic spore surface layer of *T. sanguinis,* including morphogenetic proteins required for the formation of the basement layer (SpoIVA, SpoVID, and SpoVM), coat (SafA, CotE, and CotH), and crust-like (CotY/Z[ExsY]), as well as additional structural individual proteins of each layer. (**B**) Phylogenetic tree was made with 16S ribosomal RNA gene sequence (16S rRNA) from *T. sanguinis* K031, *B. subtilis* strain 168, *B. cereus* ATCC14579, *B. anthracis* Ames Ancestor, *C. difficile* strain 630, and *C. perfringens* SM101, using ClustalW (https://www.genome.jp/tools-bin/clustalw) for their alignment. The tree was constructed using FastTree v2.1.8 with default parameters and then graphically visualized with the web tool Interactive Tree Of Life V3 (http://itol.embl.de). *B. subtilis* strain 168, *B. cereus* ATCC14579, *B. anthracis* Ames Ancestor, *C. difficile* strain 630, and *C. perfringens* SM101 genomes were downloaded from the NCBI genome database with accession number NC_000964, NZ_CP034551, NC_007530, NC_008262, and NC_009089, respectively. Phylogenetic profile shows the conservation of gene presence for relevant functional categories.

To identify the genetic basis of the spore envelope architecture inferred from microscopy, we searched the *T. sanguinis* K031 genome for homologs of coat and exosporium proteins previously characterized in *Bacillus* and *Clostridioides* species (13, 55, 80–82). At the base of the coat, we identified SpoVM (J3478_01310), a small amphipathic protein involved in coat spatial organization in *B. subtilis* spore formation (83). In addition, we identified two LysM domain-containing proteins, a SpoVID-like (J3478_14715) (84, 85) and a S-like factor (J3478_02415) (86, 87), likely implicated in early coat assembly. SpoIVA homolog (J3478_01475) was also found, which is implicated in early coat assembly (Fig. 8 and Table S6) (86–88). Together, these genes suggest that *T. sanguinis* retains conserved early morphogenetic determinants from *B. subtilis* and *C. difficile* models and are likely implicated in initiation of coat biogenesis.

Additional coat-related proteins include homologs of the *B. subtilis* morphogenetic coat protein, CotE (J3478_13570), as well as CotF (J3478_09225), CotH (J3478_15570), CotM (J3478_13295), CotQ/YvdP (J3478_11440), and CotA (J3478_15985), suggesting that *T. sanguinis* preserves much of the *B. subtilis* canonical coat repertoire (Fig. 8 and Table S6). Homologs for the tricistronic operon *cotJABC*, widely present in both Bacillus and Clostridia that encodes for spore constituents (e.g., CotJA and CotJB) and a manganese catalase (CotJC) (89, 90), were also found in *T. sanguinis* genome, CotJA (J3478_05690), two CotJB (J3478_05685 and J3478_08310), and three CotJC-like candidates (J3478_05680, J3478_05675, and J3478_08315) (Fig. 8 and Table S6). Together, these findings suggest that the spore coat of *T. sanguinis* is more similar to the *B. subtilis*-type coat composition than to the more divergent Clostridial spore coat.

Beyond the canonical coat proteins, *T. sanguinis* also encoded homologs related to exosporium-associated components. Notably, a CotY/CotZ homolog associated with the exosporium and crust layers of spores of the *B. cereus* group and *B. subtilis* (J3478_01445) (91, 92) and a CdeA homolog (J3478_01960) protein found in the exosporium of *C. difficile* (93), although its function remains unknown (Fig. 8 and Table S6). These findings suggest that *T. sanguinis* possesses not only Bacillus-like coat determinants but also proteins commonly associated with more external surface structures in Clostridial spores.

### Germination model of *T. sanguinis* spores

**Fig 9:**
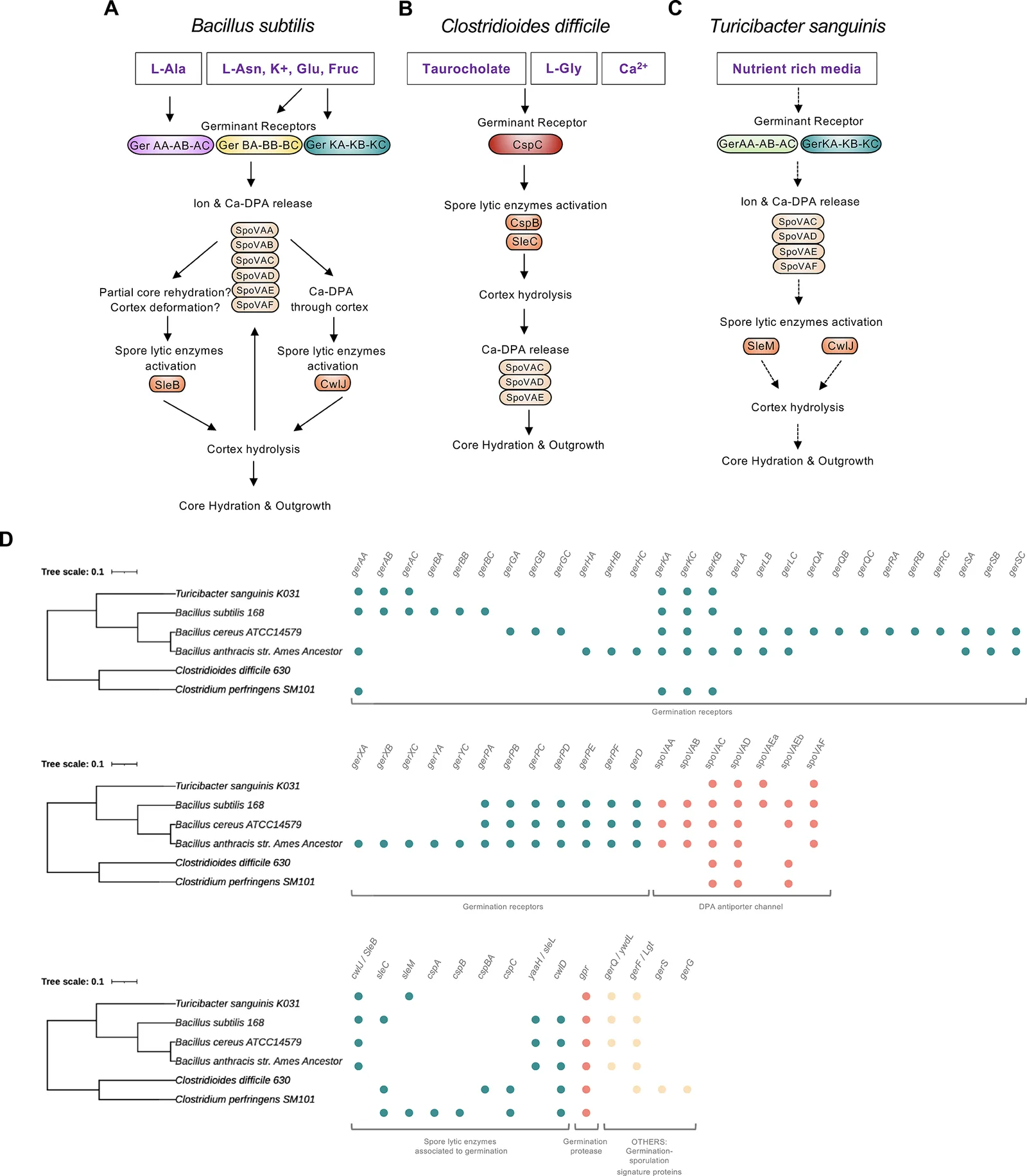
Putative germination model of *T. sanguinis* spores. Schematic spore germination signaling pathway in *B. subtilis* (**A**) and *C. difficile* (**B**) and suggested model of *T. sanguinis* (**C**), adapted from Shen et al. 2019. The germinating molecules that have been reported in *T. sanguinis* K031, *B. subtilis,* and *C. difficile* are found in white boxes, followed by their receptor, GerA, GerB, and GerK for *B. subtilis* and CspC for *C. difficile*. The suggested germination receptors of *T. sanguinis* are GerA and GerK . Proteins involved in Ca-DPA release (SpoVA) and spore lytic enzymes are shown in beige and orange cylinders. The suggested signaling pathway of *T. sanguinis* shows more similarity with the *B. subtilis* model with respect to sharing germination receptors GerA and GerK, and the spore lytic enzyme CwlJ for cortex hydrolysis. SpoVA is present in the three models. The order of hydrolysis of the cortex and DPA release via SpoVA is different in both models, and it is unknown what could be the order during the germination of spores of *T. sanguinis*. The figure was modified/adapted from Shen et al. 2019 (68). (**B**) The phylogenetic tree was made with 16S ribosomal RNA gene sequence (16S rRNA) from *T. sanguinis* K031, *B. subtilis* strain 168, *B. cereus* ATCC14579, *B. anthracis* Ames Ancestor, *C. difficile* strain 630, and *C. perfringens* SM101 using ClustalW (https://www.genome.jp/tools-bin/clustalw) for their alignment. The tree was constructed using FastTree v2.1.8 with default parameters and then graphically visualized with the web tool Interactive Tree Of Life V3 (http://itol.embl.de). *B. subtilis* strain 168, *B. cereus* ATCC14579, *B. anthracis* Ames Ancestor, *C. difficile* strain 630, and *C. perfringens* SM101 genomes were downloaded from the NCBI genome database with accession number NC_000964, NZ_CP034551, NC_007530, NC_008262, and NC_009089, respectively. The phylogenetic profile shows the conservation of gene presence for relevant functional categories.

Genomic analysis of *T. sanguinis* K031 identified two tricistronic germinant receptor operons, *gerAABC* (J3478_03110–03120) and *gerKBAC* (J3478_03230–03220) (Fig. 9C and D and Fig. S16 and Table S6), consistent with the presence of nutrient-responsive germinant receptors (13, 56, 59, 94, 95). The genome also encoded four SpoVA proteins (25, 48, 49, 96–99), SpoVAC, SpoVAD, SpoVAE, and SpoVAF (J3478_01565, J3478_01560, J3478_01555, and J3478_01550), organized as a tetracistronic operon (Fig. 9C and D and Fig. S17B and Table S6), indicating conservation of the SpoVA channel responsible for Ca-DPA release during germination.

No homologs of *C. difficile* Csp proteins or the SleC cortex hydrolase were detected, suggesting that *T. sanguinis* does not germinate in response to bile salts, glycine, and/or calcium in a Csp-dependent manner (56, 100, 101). However, *T. sanguinis* encoded orthologs of the *C. perfringens* SleM hydrolase (J3478_03615) and the *B. subtilis* cortex-lytic enzyme CwlJ (J3478_08195) and GerQ (J3478_08200) involved in CwlJ coat localization (Fig. 9C and D and Fig. S17A and S18B and C and Table S6). Two additional spore germination proteins were also found: GerF (J3478_10960) and Gpr (J3478_07110) (Fig. 9D and Fig. S18A and D and Table S6). Together, these findings indicate that *T. sanguinis* possesses a germination genetic composition like *B. subtilis* and other nutrient-germinating Bacilli rather than the bile-salt–responsive germination system of *C. difficile*.

## DISCUSSION

The gut microbiota has profound implications for human health, and perturbations in its composition can transiently or permanently deplete beneficial commensals (11). Among the diverse gut ecosystem, *T. sanguinis*, a member of the *Turicibacteraceae* family, represents an intriguing commensal organism. Its abundance has been reported to increase in Alzheimer’s disease (16–20) and has been linked to alterations in host bile acid metabolism (15, 21–23). Notably, *T. sanguinis* has also been suggested to modulate intestinal serotonin levels, with potential implications for the gut-brain axis communications (15). Nearly 40% of gut microbiota genera have the potential to form spores, highlighting sporulation as a key mechanism for persistence, both within and outside of the host environment (12, 13). Most bacterial spores studied to date belong to the class of Bacilli and Clostridia. In this context, our study expands understanding of *T. sanguinis* sporulation and spore physiology, providing detailed insight into its encoded sporulation and germination machinery. Furthermore, we enrich the repertoire of spore ultrastructural studies within the *Turicibacteraceae* family and to the broader and understudied Erysipelotrichia class, by presenting the first comprehensive ultrastructural analysis of *T. sanguinis* spores.

A major contribution of this work is the demonstration that *T. sanguinis* forms heat-resistant spores similarly to the phylogenetically distant lineages of model spore formers. Unlike the well-studied spore-former *B. subtilis*, *T. sanguinis* exhibited asynchronous sporulation, resembling the pattern observed in *C. difficile* (13, 56, 102), and was highly efficient, reaching >80% sporulation on solid medium after 48 h. The resulting spores displayed a characteristic spherical morphology with a diameter of ~ 1.1 µm, distinct from the more elongated spores of Bacilli and Clostridia species (57, 103, 104). Although *T. sanguinis* spores were able to withstand up to 60°C for 30 min without loss of viability, suggesting only mild heat resistance compared with species *C. difficile* or *C. perfringens* with highly resistant spores that can survive over 70°C and 100°C, respectively (57, 105, 106), their ability to withstand elevated temperatures with limited loss of viability indicates adaptation to survival outside the host. This level of heat tolerance, together with efficient sporulation on solid substrates, supports the idea that *T. sanguinis* can persist in environmental reservoirs and be transmitted between hosts via spore-mediated routes, adding an important ecological dimension to its role as a gut commensal and potentially contributing to the spread of health (10). Overall, these observations warrant further investigation into the role of *T. sanguinis* spores in its life cycle and host colonization dynamics.

Transmission electron microscopy revealed that the architecture of *T. sanguinis* spores shares similar canonical features observed in *C. difficile* and *B. subtilis* (55, 56), but with distinct differences in the outermost layers. In *T. sanguinis*, we identified a dehydrated core densely packed with ribosomes, surrounded by an inner membrane, a germ cell wall, and a spore-specific peptidoglycan cortex resembling those of well-characterized endospores (55). However, *T. sanguinis* spores displayed striking differences in the outer layers compared with *C. difficile* and *B. subtilis*. The inner coat of *B. subtilis* spores is approximately 75 nm thick, lamellar, and lightly stained in TEM micrographs (107), whereas *T. sanguinis* exhibited a thinner (13–58 nm) inner coat lacking lamellar organization but presenting prominent ridge-like structural features. Although similar ridge-like patterns have been reported in *Bacillus subtilis* (108), the more organized and uniform ridges observed in *T. sanguinis* may reflect lineage-specific architectural adaptations, possibly reflecting multiple initiation, termination sites of coat assembly, or specialized regions of structural reinforcement.

A striking observation in *T. sanguinis* spores is the presence of two distinct outer-layer morphotypes, thin and thick electron-dense outer coat layer spores, each following a normal distribution and independent of overall spore size. In addition, the inner coat comprises two distinguishable sublayers, with the innermost layer displaying regularly spaced ridge-like structures across the entire surface, a unique architecture not observed in *B. subtilis* or *C. difficile* spores (56, 107). To our knowledge, this represents the first report of simultaneous formation of thick and thin coat-layer phenotypes within a single bacterial population during sporulation. Moreover, the bimodality of the electron-dense outer coat layer is similar to reports in *C. difficile*, where spores exhibit thin or thick exosporium layers (13, 44, 45, 102). As in *C. difficile*, electron-dense outer coat thickness in *T. sanguinis* was independent of whole-spore diameter, reinforcing the idea that its formation is governed by dedicated regulatory mechanisms distinct from those controlling global spore morphogenesis. Notably, the drastic change in proportion of thick spores in both replicates suggests that regulation of this phenotype is variable in nature. In *C. difficile*, bimodal exosporium layers have been associated with differences in tropism toward host molecules such as E-cadherin, fibronectin, and vitronectin (109, 110), contributing to functional diversification within the spore population. Similarly, the presence of both thin and thick electron-dense outer coat morphotypes in *T. sanguinis* might reflect a regulated developmental program rather than stochastic variation in coat assembly that contributes to functionally specialized spore populations in *T. sanguinis*. Future comparative proteomic and genetic studies will be necessary to determine whether the thin and thick electron-dense outer coat morphotypes of *T. sanguinis* correspond to functionally specialized subpopulations, as observed in *C. difficile* (13, 44, 45, 102).

Another contribution is that the outermost layer of *T. sanguinis* spores displayed an amorphous appearance reminiscent of the crust found in *B. subtilis* spores (46), which surrounds the underlying electron-dense outer coat layer. Its molecular composition appears to differ from that of the *B. subtilis* crust, as *T. sanguinis* spores did not exhibit a halo when stained with India ink (Fig. 3B), a hallmark of the polysaccharide-rich crust in *B. subtilis* (46). To date, two types of outermost structures have been described surrounding the spore coat: (i) the amorphous “crust” layer in *B. subtilis* (46, 108) and (ii) the exosporium, characterized by its hair-like projections, and, in some cases, a distinct interspace separating it from the underlying coat, as observed in *B. anthracis*, *B. megaterium*, and *C. difficile* (56). The organization of the crust as a double-layered in *T. sanguinis* expands the known diversity of spore envelopes. Ongoing work in our laboratory aims to identify the molecular components and regulatory pathways governing the assembly of these outer layers.

The genomic features underlying sporulation in *T. sanguinis* reveal that this organism employs a regulatory logic that blends elements of both *Bacillus*- and *Clostridium*-like developmental systems. Although *T. sanguinis* initiates sporulation efficiently, its genome lacks the hallmark *Bacillus*-type phosphorelay components, including Spo0F, Spo0B, and the KinA–E kinases, indicating that the classical relay is not required to activate Spo0A. Instead, the presence of Spo0A-inhibitory phosphatases (Spo0E and a PtpA orthologue) suggests a regulatory strategy more closely aligned with Clostridia, where Spo0A phosphorylation is controlled directly rather than through multistep phosphotransfer (65, 111–114). This hybrid architecture highlights an evolutionary divergence in how early sporulation decisions are regulated within the Erysipelotrichia.

Despite differences in initiation, downstream elements of the sporulation program appear highly conserved. The presence of all four sporulation sigma factors (σ^F^, σ^E^, σ^G^, and σ^K^), along with their corresponding regulatory proteins, suggests that the compartmentalization of transcription between the forespore and mother cell in *T. sanguinis* follows the classical Bacilli framework (65, 115–117). Genes associated with engulfment and intercompartment communication—such as *spoIIP*, *spoIID*, *spoIIM*, *spoIIIQ*, and *gerM*—further support conservation of mid-developmental morphogenesis (65, 67). Notably, however, only the late feed-forward loops (σ^G^/SpoVT and σ^K^ /GerE) appear to be retained, whereas earlier regulatory modules typical of Bacilli (σ^F^/RsfA, σ^E^/SpoIIID, and σ^E^/GerR) were not detected. This reduction of early network complexity may reflect lineage-specific streamlining of transcriptional control.

Regulation of σ^K^ provides another key point of divergence. While *C. difficile* employs a simplified system in which σ^K^ is active upon translation (67, 79), *T. sanguinis* encodes BofA, SpoIVB, and the SpoIVFB protease, suggesting that σ^K^ activation depends on proteolytic processing of pro-σ^K^, as seen in Bacilli (65). Although early events resemble Clostridia models, later steps in coat formation and maturation appear to rely on Bacilli-like mechanisms. Together, these features indicate that *T. sanguinis* integrates regulatory strategies from both classical endospore formers, providing a unique evolutionary perspective on sporulation within the Erysipelotrichia and raising important questions about how these mixed systems are coordinated during development.

The genomic features underlying the spore surface architecture of *T. sanguinis* further support the conclusion that this organism predominantly retains a *B. subtilis*-like surface-layer structure. In particular, we identified homologs of the morphogenetic coat protein CotE, together with CotF, CotH, CotQ/YvdP, CotM, and CotA, all of which are classically associated with Bacillus-type coat assembly and maturation (81, 82, 118). The detection of the tricistronic *cotJABC* operon homologs, including CotJA, two CotJB, and multiple CotJC-like candidates, further reinforces this interpretation, as these proteins are conserved among Bacilli and Clostridia (13, 56, 81). Together, these results suggest that the spore coat composition of *T. sanguinis* is likely to be similar to the *B. subtilis* model rather than to Clostridial. Similarly, identification of a CotY/CotZ homolog, which is associated with exosporium and crust in spores of the *B. cereus* group and *B. subtilis*, respectively, and a CdeA homolog associated with the *C. difficile* exosporium suggests that *T. sanguinis* also retains proteins linked to outermost surfaces (91–93). Thus, while the core coat architecture appears *Bacillus*-like, *T. sanguinis* may have elaborated this structure with additional components that contribute to the crust-like outer layer and the observed electron-dense outer coat heterogeneity. This raises several questions on both the mechanism of assembly of these layers and their implication in the biology of *T. sanguinis* spores.

The genomic and phenotypic characteristics of *T. sanguinis* suggest that its germination mechanism is more closely aligned with Bacilli-like pathways than with those of Clostridia. The presence of two complete tricistronic Ger receptor operons (*gerKABC* and *gerAABC*) implies that nutrient-derived signals are key triggers for germination (59, 65, 119). Given that Ger-family receptors in *B. subtilis* recognize amino acids and sugars such as L-asparagine, D-glucose, and L-alanine (AGFK pathway) (94), *T. sanguinis* may respond to analogous small molecules, although this remains to be experimentally validated. A second hallmark of Bacilli-like germination is evident in the organization of SpoVA proteins associated with Ca-DPA transport, which is consistent with the presence of Ca-DPA in *T. sanguinis* spores. The arrangement of all SpoVA components in a single operon suggests coordinated control of Ca-DPA release, a process that typically precedes cortex hydrolysis in Bacilli (94). The predicted reliance on CwlJ- and SleM-like hydrolases further supports this interpretation, as these enzymes fulfill the cortex-lytic role played by SleB/SleC in *Bacillus* or by the CspB–SleC system in *C. difficile* (56, 65). Taken together, the genomic evidence strongly points toward a germination mechanism in which nutrient sensing triggers Ca-DPA efflux through SpoVA, followed by cortex degradation—mirroring the temporal hierarchy described for *B. subtilis* (120).

Functional assays reinforce this interpretation. The detection of ~10 µg/mL of DPA places *T. sanguinis* at the lower end of the DPA spectrum reported for well-characterized spores (121, 122), confirming that DPA sequestration is a conserved feature of its developmental program. A notable observation was that, unlike other model spore-former species (e.g., *B. subtilis* and *C. difficile*), *T. sanguinis* spores did not require heat activation to trigger rapid spore germination in nutrient-rich media (e.g., 70:30 and YCFA). Canonically, a nonlethal heat treatment of dormant spores is used to activate spores toward germinant sensing, which leads to rapid germination within less than 60 min at 37°C (59, 119). Although recent work demonstrates that the Ger-family of receptors form oligomeric membrane channels that act as nutrient-gated ion channels (95), the precise mechanism of how heat activation works is unclear, although it is thought to cause conformational changes at the receptor level. These changes are likely to render the receptors nutrient-binding ready, allowing a rapid germination response (59, 119). In this context, *T. sanguinis* spores’ ability to germinate in the absence of heat activation suggests that either the inner membrane is more fluidic or that its Ger receptors are in an active state. These observations warrant further work to understand the molecular basis of these differential requirements as well as the identity of *T. sanguinis* germinants. Although we have not addressed individual amino acid requirements for *T. sanguinis* spore germination, the fact that 70:30 and, to a lesser extent, YCFA media, but not BHIS, were able to trigger germination is intriguing. While all three nutrient broths have yeast extract, 70:30 and YCFA differ from BHIS in that they contain Bacto Casitone, Bacto peptone, and Proteose peptone digests (123). These components are enzymatic digests with a higher availability of amino acids than BHIS, suggesting that free amino acids are acting as germinants for *T. sanguinis* spores. Altogether, these findings position *T. sanguinis* among the Bacilli-like germinating taxa, despite its distant evolutionary placement within the Erysipelotrichia. The discovery of nutrient-responsive germination mechanisms, heat-activation dependence, and a Bacilli-like SpoVA-driven Ca-DPA release pathway highlights the conservation of canonical germination pathways in gut commensal.

## Data Availability

This Whole Genome Shotgun project has been deposited at DDBJ/ENA/GenBank under the accession JAGBKO000000000. The version described in this paper is version JAGBKO010000000.

## SUPPLEMENTAL MATERIAL

Supplemental figures jb.00029-26-s0001.docx Figures S1 to S18.

Table S1 jb.00029-26-s0002.xlsx Genomes used in this study and associated metadata.

Table S2 jb.00029-26-s0003.xlsx Taxonomic allocation using 16S rRNA and ribosomal protein phylogenies using phylogenetically informative markers.

Table S3 jb.00029-26-s0004.xlsx Overall genome relatedness indexes calculations of selected Turicibacter spp. inferred from nucleotide data using alignment-based methods (ANIb, dDDH AAI).

Table S4 jb.00029-26-s0005.xlsx List of genes involved in sporulation, germination, and spore structure reported to date in spore-former models *B. subtilis* 168, *B. cereus* ATCC14579, *B. anthracis* Ames Ancestor, *C. perfringens* SM101, and *C. difficile* 630.

Table S5 jb.00029-26-s0006.xlsx Output table with domain/family information of each CDS per genome of spore-former models *B. subtilis* 168, *B. cereus* ATCC14579, *B. anthracis* Ames Ancestor, *C. perfringens* SM101, and *C. difficile* 630.

Table S6 jb.00029-26-s0007.xlsx Summary of sporulation, spore structure and germination-related orthologs found in *T. sanguinis* K031 genome.

ASM does not own the copyrights to Supplemental Material that may be linked to, or accessed through, an article. The authors have granted ASM a non-exclusive, world-wide license to publish the Supplemental Material files. Please contact the corresponding author directly for reuse.

## References

[1] Hoegenauer C, Hammer HF, Mahnert A, Moissl-Eichinger C. 2022. Methanogenic archaea in the human gastrointestinal tract. Nat Rev Gastroenterol Hepatol 19:805–813. doi:10.1038/s41575-022-00673-z 36050385

[2] Miyauchi E, Shimokawa C, Steimle A, Desai MS, Ohno H. 2023. The impact of the gut microbiome on extra-intestinal autoimmune diseases. Nat Rev Immunol 23:9–23. doi:10.1038/s41577-022-00727-y 35534624

[3] Qin J, Li R, Raes J, Arumugam M, Burgdorf KS, Manichanh C, Nielsen T, Pons N, Levenez F, Yamada T, et al.. 2010. A human gut microbial gene catalogue established by metagenomic sequencing. Nature 464:59–65. doi:10.1038/nature08821 20203603 PMC3779803

[4] Almeida A, Mitchell AL, Boland M, Forster SC, Gloor GB, Tarkowska A, Lawley TD, Finn RD. 2019. A new genomic blueprint of the human gut microbiota. Nature 568:499–504. doi:10.1038/s41586-019-0965-1 30745586 PMC6784870

[5] Lynch SV, Pedersen O. 2016. The human intestinal microbiome in health and disease. N Engl J Med 375:2369–2379. doi:10.1056/NEJMra1600266 27974040

[6] Shealy NG, Yoo W, Byndloss MX. 2021. Colonization resistance: metabolic warfare as a strategy against pathogenic enterobacteriaceae. Curr Opin Microbiol 64:82–90. doi:10.1016/j.mib.2021.09.014 34688039 PMC8612973

[7] Halfvarson J, Brislawn CJ, Lamendella R, Vázquez-Baeza Y, Walters WA, Bramer LM, D’Amato M, Bonfiglio F, McDonald D, Gonzalez A, McClure EE, Dunklebarger MF, Knight R, Jansson JK. 2017. Dynamics of the human gut microbiome in inflammatory bowel disease. Nat Microbiol 2:17004. doi:10.1038/nmicrobiol.2017.4 28191884 PMC5319707

[8] Sepúlveda-Rivera V, Olivieri-Henry G, Morales-González H, Ruiz-Adames J, Herrero-Rivera C, Rentas-Echeverria A, Cardona-Berdecia V, Soler-Llompart C, Sala-Morales AC, Pérez-Montero G, Blanco-Ruiz E, Godoy-Vitorino F. 2025. Gut microbiota distinguishes aging hispanics with alzheimer’s disease: associations with cognitive impairment and severity. Sci Rep 15:28505. doi:10.1038/s41598-025-13262-2 40764790 PMC12325907

[9] López-Otín C, Blasco MA, Partridge L, Serrano M, Kroemer G. 2023. Hallmarks of aging: an expanding universe. Cell 186:243–278. doi:10.1016/j.cell.2022.11.001 36599349

[10] Browne HP, Neville BA, Forster SC, Lawley TD. 2017. Transmission of the gut microbiota: spreading of health. Nat Rev Microbiol 15:531–543. doi:10.1038/nrmicro.2017.50 28603278 PMC5837012

[11] Schwartz DJ, Langdon AE, Dantas G. 2020. Understanding the impact of antibiotic perturbation on the human microbiome. Genome Med 12:82. doi:10.1186/s13073-020-00782-x 32988391 PMC7523053

[12] Browne HP, Forster SC, Anonye BO, Kumar N, Neville BA, Stares MD, Goulding D, Lawley TD. 2016. Culturing of “unculturable” human microbiota reveals novel taxa and extensive sporulation. Nature 533:543–546. doi:10.1038/nature17645 27144353 PMC4890681

[13] Paredes-Sabja D, Cid-Rojas F, Pizarro-Guajardo M. 2022. Assembly of the exosporium layer in *Clostridioides difficile* spores. Curr Opin Microbiol 67:102137. doi:10.1016/j.mib.2022.01.008 35182899

[14] Deakin LJ, Clare S, Fagan RP, Dawson LF, Pickard DJ, West MR, Wren BW, Fairweather NF, Dougan G, Lawley TD. 2012. The *Clostridium difficile* spo0A gene is a persistence and transmission factor. Infect Immun 80:2704–2711. doi:10.1128/IAI.00147-12 22615253 PMC3434595

[15] Fung TC, Vuong HE, Luna CDG, Pronovost GN, Aleksandrova AA, Riley NG, Vavilina A, McGinn J, Rendon T, Forrest LR, Hsiao EY. 2019. Intestinal serotonin and fluoxetine exposure modulate bacterial colonization in the gut. Nat Microbiol 4:2064–2073. doi:10.1038/s41564-019-0540-4 31477894 PMC6879823

[16] Dunham SJB, McNair KA, Adams ED, Avelar-Barragan J, Forner S, Mapstone M, Whiteson KL. 2022. Longitudinal analysis of the microbiome and metabolome in the 5xfAD mouse model of alzheimer’s disease. mBio 13:e0179422. doi:10.1128/mbio.01794-22 36468884 PMC9765021

[17] Borsom EM, Conn K, Keefe CR, Herman C, Orsini GM, Hirsch AH, Palma Avila M, Testo G, Jaramillo SA, Bolyen E, Lee K, Caporaso JG, Cope EK. 2023. Predicting neurodegenerative disease using prepathology gut microbiota composition: a longitudinal study in mice modeling alzheimer’s disease pathologies. Microbiol Spectr 11:e0345822. doi:10.1128/spectrum.03458-22 36877047 PMC10101110

[18] Jin M, Li J, Liu F, Lyu N, Wang K, Wang L, Liang S, Tao H, Zhu B, Alkasir R. 2019. Analysis of the gut microflora in patients with parkinson’s disease. Front Neurosci 13:1184. doi:10.3389/fnins.2019.01184 31824239 PMC6883725

[19] Barandouzi ZA, Starkweather AR, Henderson WA, Gyamfi A, Cong XS. 2020. Altered composition of gut microbiota in depression: a systematic review. Front Psychiatry 11:541. doi:10.3389/fpsyt.2020.00541 32587537 PMC7299157

[20] Lynch JB, Gonzalez EL, Choy K, Faull KF, Jewell T, Arellano A, Liang J, Yu KB, Paramo J, Hsiao EY. 2023. Gut microbiota *Turicibacter strains* differentially modify bile acids and host lipids. Nat Commun 14:3669. doi:10.1038/s41467-023-39403-7 37339963 PMC10281990

[21] Liu W, Crott JW, Lyu L, Pfalzer AC, Li J, Choi SW, Yang Y, Mason JB, Liu Z. 2016. Diet- and genetically-induced obesity produces alterations in the microbiome, inflammation and *Wnt* pathway in the intestine of Apc^+/1638N^ mice: comparisons and contrasts. J Cancer 7:1780–1790. doi:10.7150/jca.15792 27698916 PMC5039360

[22] Li TT, Tong AJ, Liu YY, Huang ZR, Wan XZ, Pan YY, Jia RB, Liu B, Chen XH, Zhao C. 2019. Polyunsaturated fatty acids from microalgae Spirulina platensis modulates lipid metabolism disorders and gut microbiota in high-fat diet rats. Food Chem Toxicol 131:110558. doi:10.1016/j.fct.2019.06.005 31175915

[23] Velázquez KT, Enos RT, Bader JE, Sougiannis AT, Carson MS, Chatzistamou I, Carson JA, Nagarkatti PS, Nagarkatti M, Murphy EA. 2019. Prolonged high-fat-diet feeding promotes non-alcoholic fatty liver disease and alters gut microbiota in mice. World J Hepatol 11:619–637. doi:10.4254/wjh.v11.i8.619 31528245 PMC6717713

[24] Harwood CR, Cutting SM. 1990. Molecular biological methods for Bacillus. Wiley, Chichester ; New York.

[25] Paredes-Sabja D, Setlow B, Setlow P, Sarker MR. 2008. Characterization of *Clostridium perfringens* spores that lack SpoVA proteins and dipicolinic acid. J Bacteriol 190:4648–4659. doi:10.1128/JB.00325-08 18469104 PMC2446781

[26] Bolger AM, Lohse M, Usadel B. 2014. Trimmomatic: a flexible trimmer for illumina sequence data. Bioinformatics 30:2114–2120. doi:10.1093/bioinformatics/btu170 24695404 PMC4103590

[27] Koren S, Rhie A, Walenz BP, Dilthey AT, Bickhart DM, Kingan SB, Hiendleder S, Williams JL, Smith TPL, Phillippy AM. 2018. De novo assembly of haplotype-resolved genomes with trio binning. Nat Biotechnol 36:1174–1182. doi:10.1038/nbt.4277 PMC6476705 30346939

[28] Antipov D, Korobeynikov A, McLean JS, Pevzner PA. 2016. hybridSPAdes: an algorithm for hybrid assembly of short and long reads. Bioinformatics 32:1009–1015. doi:10.1093/bioinformatics/btv688 26589280 PMC4907386

[29] Langmead B, Salzberg SL. 2012. Fast gapped-read alignment with bowtie 2. Nat Methods 9:357–359. doi:10.1038/nmeth.1923 22388286 PMC3322381

[30] Tatusova T, DiCuccio M, Badretdin A, Chetvernin V, Nawrocki EP, Zaslavsky L, Lomsadze A, Pruitt KD, Borodovsky M, Ostell J. 2016. Ncbi prokaryotic genome annotation pipeline. Nucleic Acids Res 44:6614–6624. doi:10.1093/nar/gkw569 27342282 PMC5001611

[31] Raes J, Korbel JO, Lercher MJ, von Mering C, Bork P. 2007. Prediction of effective genome size in metagenomic samples. Genome Biol 8:R10. doi:10.1186/gb-2007-8-1-r10 17224063 PMC1839125

[32] Li W, Godzik A. 2006. Cd-hit: a fast program for clustering and comparing large sets of protein or nucleotide sequences. Bioinformatics 22:1658–1659. doi:10.1093/bioinformatics/btl158 16731699

[33] Camacho C, Coulouris G, Avagyan V, Ma N, Papadopoulos J, Bealer K, Madden TL. 2009. BLAST+: architecture and applications. BMC Bioinformatics 10:421. doi:10.1186/1471-2105-10-421 20003500 PMC2803857

[34] Guindon S, Dufayard JF, Lefort V, Anisimova M, Hordijk W, Gascuel O. 2010. New algorithms and methods to estimate maximum-likelihood phylogenies: assessing the performance of PhyML 3.0. Syst Biol 59:307–321. doi:10.1093/sysbio/syq010 20525638

[35] Howe K, Bateman A, Durbin R. 2002. QuickTree: building huge neighbour-joining trees of protein sequences. Bioinformatics 18:1546–1547. doi:10.1093/bioinformatics/18.11.1546 12424131

[36] Price MN, Dehal PS, Arkin AP. 2009. FastTree: computing large minimum evolution trees with profiles instead of a distance matrix. Mol Biol Evol 26:1641–1650. doi:10.1093/molbev/msp077 19377059 PMC2693737

[37] Katoh K, Standley DM. 2013. Mafft multiple sequence alignment software version 7: improvements in performance and usability. Mol Biol Evol 30:772–780. doi:10.1093/molbev/mst010 23329690 PMC3603318

[38] Lefort V, Desper R, Gascuel O. 2015. FastME 2.0: a comprehensive, accurate, and fast distance-based phylogeny inference program. Mol Biol Evol 32:2798–2800. doi:10.1093/molbev/msv150 26130081 PMC4576710

[39] Huelsenbeck JP, Ronquist F. 2001. Mrbayes: bayesian inference of phylogenetic trees. Bioinformatics 17:754–755. doi:10.1093/bioinformatics/17.8.754 11524383

[40] Richter M, Rosselló-Móra R. 2009. Shifting the genomic gold standard for the prokaryotic species definition. Proc Natl Acad Sci USA 106:19126–19131. doi:10.1073/pnas.0906412106 19855009 PMC2776425

[41] Pritchard L, Glover RH, Humphris S, Elphinstone JG, Toth IK. 2016. Genomics and taxonomy in diagnostics for food security: soft-rotting enterobacterial plant pathogens. Anal Methods 8:12–24. doi:10.1039/C5AY02550H

[42] Meier-Kolthoff JP, Klenk HP, Göker M. 2014. Taxonomic use of DNA G+C content and DNA-DNA hybridization in the genomic age. Int J Syst Evol Microbiol 64:352–356. doi:10.1099/ijs.0.056994-0 24505073

[43] Meier-Kolthoff JP, Auch AF, Klenk HP, Göker M. 2013. Genome sequence-based species delimitation with confidence intervals and improved distance functions. BMC Bioinformatics 14:60. doi:10.1186/1471-2105-14-60 23432962 PMC3665452

[44] Pizarro-Guajardo M, Calderón-Romero P, Paredes-Sabja D. 2016. Ultrastructure variability of the exosporium layer of *Clostridium difficile* spores from sporulating cultures and biofilms. Appl Environ Microbiol 82:5892–5898. doi:10.1128/AEM.01463-16 27474709 PMC5038037

[45] Pizarro-Guajardo Marjorie, Calderón-Romero P, Castro-Córdova P, Mora-Uribe P, Paredes-Sabja D. 2016. Ultrastructural variability of the exosporium layer of *Clostridium difficile* spores. Appl Environ Microbiol 82:2202–2209. doi:10.1128/AEM.03410-15 26850296 PMC4807528

[46] Shuster B, Khemmani M, Abe K, Huang X, Nakaya Y, Maryn N, Buttar S, Gonzalez AN, Driks A, Sato T, Eichenberger P. 2019. Contributions of crust proteins to spore surface properties in *Bacillus subtilis*. Mol Microbiol 111:825–843. doi:10.1111/mmi.14194 30582883 PMC6417949

[47] Skinner SO, Sepúlveda LA, Xu H, Golding I. 2013. Measuring mRNA copy number in individual *Escherichia coli* cells using single-molecule fluorescent in situ hybridization. Nat Protoc 8:1100–1113. doi:10.1038/nprot.2013.066 23680982 PMC4029592

[48] Francis Michael B, Sorg JA. 2016. Dipicolinic acid release by germinating *Clostridium difficile* spores occurs through a mechanosensing mechanism. mSphere 1:e00306-16. doi:10.1128/mSphere.00306-16 27981237 PMC5156672

[49] Francis M.B, Allen CA, Sorg JA. 2015. Spore cortex hydrolysis precedes dipicolinic acid release during *Clostridium difficile* spore germination. J Bacteriol 197:2276–2283. doi:10.1128/JB.02575-14 25917906 PMC4524186

[50] Pol IE, van Arendonk WG, Mastwijk HC, Krommer J, Smid EJ, Moezelaar R. 2001. Sensitivities of germinating spores and carvacrol-adapted vegetative cells and spores of *Bacillus cereus* to nisin and pulsed-electric-field treatment. Appl Environ Microbiol 67:1693–1699. doi:10.1128/AEM.67.4.1693-1699.2001 11282623 PMC92787

[51] Hug LA, Baker BJ, Anantharaman K, Brown CT, Probst AJ, Castelle CJ, Butterfield CN, Hernsdorf AW, Amano Y, Ise K, Suzuki Y, Dudek N, Relman DA, Finstad KM, Amundson R, Thomas BC, Banfield JF. 2016. A new view of the tree of life. Nat Microbiol 1:16048. doi:10.1038/nmicrobiol.2016.48 27572647

[52] Bosshard PP, Zbinden R, Altwegg M. 2002. *Turicibacter sanguinis* gen. nov., sp. nov., a novel anaerobic, gram-positive bacterium. Int J Syst Evol Microbiol 52:1263–1266. doi:10.1099/00207713-52-4-1263 12148638

[53] Maki JJ, Looft T. 2022. *Turicibacter bilis* sp. nov., a novel bacterium isolated from the chicken eggshell and swine ileum. Int J Syst Evol Microbiol 72:005153. doi:10.1099/ijsem.0.005153 35084297 PMC8895650

[54] Waller LN, Stump MJ, Fox KF, Harley WM, Fox A, Stewart GC, Shahgholi M. 2005. Identification of a second collagen-like glycoprotein produced by *Bacillus anthracis* and demonstration of associated spore-specific sugars. J Bacteriol 187:4592–4597. doi:10.1128/JB.187.13.4592-4597.2005 15968070 PMC1151769

[55] Henriques AO, Moran CP Jr. 2007. Structure, assembly, and function of the spore surface layers. Annu Rev Microbiol 61:555–588. doi:10.1146/annurev.micro.61.080706.093224 18035610

[56] Paredes-Sabja D, Shen A, Sorg JA. 2014. *Clostridium difficile* spore biology: sporulation, germination, and spore structural proteins. Trends Microbiol 22:406–416. doi:10.1016/j.tim.2014.04.003 24814671 PMC4098856

[57] Novak JS, Juneja VK, McClane BA. 2003. An ultrastructural comparison of spores from various strains of *Clostridium perfringens* and correlations with heat resistance parameters. Int J Food Microbiol 86:239–247. doi:10.1016/s0168-1605(02)00550-0 12915035

[58] Baloh M, Sorg JA. 2021. Clostridioides difficile SpoVAD and SpoVAE interact and are required for DPA uptake into spores. J Bacteriol Jb0039421. doi:10.1101/2021.07.28.454260 PMC8508128 34424035

[59] Paredes-Sabja D, Setlow P, Sarker MR. 2011. Germination of spores of bacillales and clostridiales species: mechanisms and proteins involved. Trends Microbiol 19:85–94. doi:10.1016/j.tim.2010.10.004 21112786

[60] Shen A, Fimlaid KA, Pishdadian K. 2016. Inducing and quantifying *Clostridium difficile* spore formation. Methods Mol Biol 1476:129–142. doi:10.1007/978-1-4939-6361-4_10 27507338

[61] Giel JL, Sorg JA, Sonenshein AL, Zhu J. 2010. Metabolism of bile salts in mice influences spore germination in *Clostridium difficile*. PLoS One 5:e8740. doi:10.1371/journal.pone.0008740 20090901 PMC2806926

[62] Edwards AN, Wetzel D, DiCandia MA, McBride SM. 2022. Three orphan histidine kinases inhibit clostridioides difficile sporulation. J Bacteriol 204:e0010622. doi:10.1128/jb.00106-22 35416689 PMC9112875

[63] Mutlu A, Kaspar C, Becker N, Bischofs IB. 2020. A spore quality-quantity tradeoff favors diverse sporulation strategies in *Bacillus subtilis*. ISME J 14:2703–2714. doi:10.1038/s41396-020-0721-4 32724142 PMC7784978

[64] DiCandia MA, Edwards AN, Alcaraz YB, Monteiro MP, Lee CD, Vargas Cuebas G, Bagchi P, McBride SM. 2024. A conserved switch controls virulence, sporulation, and motility in *C. difficile*. PLoS Pathog 20:e1012224. doi:10.1371/journal.ppat.1012224 38739653 PMC11115286

[65] Shen A, Edwards AN, Sarker MR, Paredes-Sabja D. 2019. Sporulation and germination in clostridial pathogens. Microbiol Spectr 7. doi:10.1128/microbiolspec.GPP3-0017-2018 PMC6927485 31858953

[66] Tan IS, Ramamurthi KS. 2014. Spore formation in *Bacillus subtilis*. Environ Microbiol Rep 6:212–225. doi:10.1111/1758-2229.12130 24983526 PMC4078662

[67] Al-Hinai MA, Jones SW, Papoutsakis ET. 2015. The *Clostridium* sporulation programs: diversity and preservation of endospore differentiation. Microbiol Mol Biol Rev 79:19–37. doi:10.1128/MMBR.00025-14 25631287 PMC4402964

[68] Fimlaid KA, Shen A. 2015. Diverse mechanisms regulate sporulation sigma factor activity in the firmicutes. Curr Opin Microbiol 24:88–95. doi:10.1016/j.mib.2015.01.006 25646759 PMC4380625

[69] Londoño-Vallejo JA, Stragier P. 1995. Cell-cell signaling pathway activating a developmental transcription factor in *Bacillus subtilis*. Genes Dev 9:503–508. doi:10.1101/gad.9.4.503 7883171

[70] Eichenberger P, Jensen ST, Conlon EM, van Ooij C, Silvaggi J, González-Pastor JE, Fujita M, Ben-Yehuda S, Stragier P, Liu JS, Losick R. 2003. The sigmaE regulon and the identification of additional sporulation genes in *Bacillus subtilis*. J Mol Biol 327:945–972. doi:10.1016/s0022-2836(03)00205-5 12662922

[71] Frandsen N, Stragier P. 1995. Identification and characterization of the *Bacillus subtilis* spoIIP locus. J Bacteriol 177:716–722. doi:10.1128/jb.177.3.716-722.1995 7836306 PMC176648

[72] Rodrigues CDA, Ramírez-Guadiana FH, Meeske AJ, Wang X, Rudner DZ. 2016. GerM is required to assemble the basal platform of the SpoIIIA-SpoIIQ transenvelope complex during sporulation in *Bacillus subtilis*. Mol Microbiol 102:260–273. doi:10.1111/mmi.13457 27381174 PMC5055438

[73] Meisner J, Wang X, Serrano M, Henriques AO, Moran CP Jr. 2008. A channel connecting the mother cell and forespore during bacterial endospore formation. Proc Natl Acad Sci USA 105:15100–15105. doi:10.1073/pnas.0806301105 18812514 PMC2567499

[74] Doan T, Morlot C, Meisner J, Serrano M, Henriques AO, Moran CP, Rudner DZ. 2009. Novel secretion apparatus maintains spore integrity and developmental gene expression in *Bacillus subtilis*. PLoS Genet 5:e1000566. doi:10.1371/journal.pgen.1000566 19609349 PMC2703783

[75] Camp AH, Losick R. 2009. A feeding tube model for activation of a cell-specific transcription factor during sporulation in *Bacillus subtilis*. Genes Dev 23:1014–1024. doi:10.1101/gad.1781709 19390092 PMC2675869

[76] Morlot C, Uehara T, Marquis KA, Bernhardt TG, Rudner DZ. 2010. A highly coordinated cell wall degradation machine governs spore morphogenesis in *Bacillus subtilis*. Genes Dev 24:411–422. doi:10.1101/gad.1878110 20159959 PMC2816739

[77] Gutierrez J, Smith R, Pogliano K. 2010. SpoIID-mediated peptidoglycan degradation is required throughout engulfment during *Bacillus subtilis* sporulation. J Bacteriol 192:3174–3186. doi:10.1128/JB.00127-10 20382772 PMC2901704

[78] Chastanet A, Losick R. 2007. Engulfment during sporulation in *Bacillus subtilis* is governed by a multi-protein complex containing tandemly acting autolysins. Mol Microbiol 64:139–152. doi:10.1111/j.1365-2958.2007.05652.x 17376078

[79] de Hoon MJL, Eichenberger P, Vitkup D. 2010. Hierarchical evolution of the bacterial sporulation network. Curr Biol 20:R735–45. doi:10.1016/j.cub.2010.06.031 20833318 PMC2944226

[80] McKenney PT, Driks A, Eskandarian HA, Grabowski P, Guberman J, Wang KH, Gitai Z, Eichenberger P. 2010. A distance-weighted interaction map reveals a previously uncharacterized layer of the *Bacillus subtilis* spore coat. Curr Biol 20:934–938. doi:10.1016/j.cub.2010.03.060 20451384 PMC2920530

[81] Driks A, Eichenberger P. 2016. The spore coat. Microbiol Spectr 4. doi:10.1128/microbiolspec.TBS-0023-2016 27227299

[82] McKenney PT, Driks A, Eichenberger P. 2013. The *Bacillus subtilis* endospore: assembly and functions of the multilayered coat. Nat Rev Microbiol 11:33–44. doi:10.1038/nrmicro2921 23202530 PMC9910062

[83] Cutting S, Anderson M, Lysenko E, Page A, Tomoyasu T, Tatematsu K, Tatsuta T, Kroos L, Ogura T. 1997. SpoVM, a small protein essential to development in *Bacillus subtilis*, interacts with the ATP-dependent protease FtsH. J Bacteriol 179:5534–5542. doi:10.1128/jb.179.17.5534-5542.1997 9287010 PMC179426

[84] Wang KH, Isidro AL, Domingues L, Eskandarian HA, McKenney PT, Drew K, Grabowski P, Chua MH, Barry SN, Guan M, Bonneau R, Henriques AO, Eichenberger P. 2009. The coat morphogenetic protein SpoVID is necessary for spore encasement in *Bacillus subtilis*. Mol Microbiol 74:634–649. doi:10.1111/j.1365-2958.2009.06886.x 19775244 PMC2806667

[85] Ozin AJ, Henriques AO, Yi H, Moran CP Jr. 2000. Morphogenetic proteins SpoVID and SafA form a complex during assembly of the *Bacillus subtilis* spore coat . J Bacteriol 182:1828–1833. doi:10.1128/JB.182.7.1828-1833.2000 10714986 PMC101864

[86] Touchette MH, Benito de la Puebla H, Ravichandran P, Shen A. 2019. SpoIVA-SipL complex formation is essential for *Clostridioides difficile* spore assembly. J Bacteriol 201. doi:10.1128/JB.00042-19 PMC6436350 30692174

[87] Putnam EE, Nock AM, Lawley TD, Shen A. 2013. SpoIVA and SipL are *Clostridium difficile* spore morphogenetic proteins. J Bacteriol 195:1214–1225. doi:10.1128/JB.02181-12 23292781 PMC3592010

[88] Ramamurthi KS, Losick R. 2008. ATP-driven self-assembly of a morphogenetic protein in *Bacillus subtilis*. Mol Cell 31:406–414. doi:10.1016/j.molcel.2008.05.030 18691972 PMC2585998

[89] Seyler RW Jr, Henriques AO, Ozin AJ, Moran CP Jr. 1997. Assembly and interactions of cotJ-encoded proteins, constituents of the inner layers of the *Bacillus subtilis* spore coat. Mol Microbiol 25:955–966. doi:10.1111/j.1365-2958.1997.mmi532.x 9364920

[90] Permpoonpattana P, Phetcharaburanin J, Mikelsone A, Dembek M, Tan S, Brisson M-C, La Ragione R, Brisson AR, Fairweather N, Hong HA, Cutting SM. 2013. Functional characterization of *Clostridium difficile* spore coat proteins. J Bacteriol 195:1492–1503. doi:10.1128/JB.02104-12 23335421 PMC3624542

[91] Durand-Heredia J, Stewart GC. 2022. Localization of the CotY and ExsY proteins to the exosporium basal layer of *Bacillus anthracis*. Microbiologyopen 11:e1327. doi:10.1002/mbo3.1327 36314748 PMC9562818

[92] Johnson MJ, Todd SJ, Ball DA, Shepherd AM, Sylvestre P, Moir A. 2006. ExsY and CotY are required for the correct assembly of the exosporium and spore coat of *Bacillus cereus*. J Bacteriol 188:7905–7913. doi:10.1128/JB.00997-06 16980471 PMC1636315

[93] Díaz-González F, Milano M, Olguin-Araneda V, Pizarro-Cerda J, Castro-Córdova P, Tzeng S-C, Maier CS, Sarker MR, Paredes-Sabja D. 2015. Protein composition of the outermost exosporium-like layer of *Clostridium difficile* 630 spores. J Proteomics 123:1–13. doi:10.1016/j.jprot.2015.03.035 25849250 PMC6764588

[94] Setlow P, Wang S, Li YQ. 2017. Germination of spores of the orders bacillales and clostridiales. Annu Rev Microbiol 71:459–477. doi:10.1146/annurev-micro-090816-093558 28697670

[95] Gao Y, Amon JD, Artzi L, Ramírez-Guadiana FH, Brock KP, Cofsky JC, Marks DS, Kruse AC, Rudner DZ. 2023. Bacterial spore germination receptors are nutrient-gated ion channels. Science 380:387–391. doi:10.1126/science.adg9829 37104613 PMC11154005

[96] Gao Y, Barajas-Ornelas RDC, Amon JD, Ramírez-Guadiana FH, Alon A, Brock KP, Marks DS, Kruse AC, Rudner DZ. 2022. The SpoVA membrane complex is required for dipicolinic acid import during sporulation and export during germination. Genes Dev 36:634–646. doi:10.1101/gad.349488.122 35654455 PMC9186386

[97] Vepachedu VR, Setlow P. 2005. Localization of SpoVAD to the inner membrane of spores of *Bacillus subtilis*. J Bacteriol 187:5677–5682. doi:10.1128/JB.187.16.5677-5682.2005 16077113 PMC1196082

[98] Tovar-Rojo F, Chander M, Setlow B, Setlow P. 2002. The products of the spoVA operon are involved in dipicolinic acid uptake into developing spores of *Bacillus subtilis*. J Bacteriol 184:584–587. doi:10.1128/JB.184.2.584-587.2002 11751839 PMC139579

[99] Donnelly ML, Fimlaid KA, Shen A. 2016. Characterization of *Clostridium difficile* spores lacking either SpoVAC or dipicolinic acid synthetase. J Bacteriol 198:1694–1707. doi:10.1128/JB.00986-15 27044622 PMC4959285

[100] Kochan TJ, Somers MJ, Kaiser AM, Shoshiev MS, Hagan AK, Hastie JL, Giordano NP, Smith AD, Schubert AM, Carlson PE, Hanna PC. 2017. Intestinal calcium and bile salts facilitate germination of *Clostridium difficile* spores. PLoS Pathog 13:e1006443. doi:10.1371/journal.ppat.1006443 28704538 PMC5509370

[101] Ribis JW, Ravichandran P, Putnam EE, Pishdadian K, Shen A. 2017. The conserved spore coat protein SpoVM Is largely dispensable in *Clostridium difficile* spore formation. mSphere 2:e00315-17. doi:10.1128/mSphere.00315-17 PMC5607322 28959733

[102] Pizarro-Guajardo M, Calderón-Romero P, Romero-Rodríguez A, Paredes-Sabja D. 2020. Characterization of exosporium layer variability of *Clostridioides difficile* spores in the epidemically relevant strain R20291. Front Microbiol 11:1345. doi:10.3389/fmicb.2020.01345 32714296 PMC7343902

[103] Antunes W, Pereira FC, Feliciano C, Saujet L, Td V, Couture-Tosi E, Pechine S, Bruxelle J-F, Janoir C, Melo L, Brito P, Martin-Verstraete I, Serrano M, Dupuy B, Henriques AO. 2018. Structure and assembly of a *Clostridioides Difficile* spore polar appendage. bioRxiv

[104] Carrera M, Zandomeni RO, Fitzgibbon J, Sagripanti JL. 2007. Difference between the spore sizes of *Bacillus anthracis* and other *Bacillus species*. J Appl Microbiol 102:303–312. doi:10.1111/j.1365-2672.2006.03111.x 17241334

[105] Rodriguez-Palacios A, Lejeune JT. 2011. Moist-heat resistance, spore aging, and superdormancy in *Clostridium difficile*. Appl Environ Microbiol 77:3085–3091. doi:10.1128/AEM.01589-10 21398481 PMC3126382

[106] Raju D, Sarker MR. 2005. Comparison of the levels of heat resistance of wild-type, cpe knockout, and cpe plasmid-cured *Clostridium perfringens* type a strains. Appl Environ Microbiol 71:7618–7620. doi:10.1128/AEM.71.11.7618-7620.2005 16269817 PMC1287616

[107] Driks A. 1999. *Bacillus subtilis* spore coat. Microbiol Mol Biol Rev 63:1–20. doi:10.1128/MMBR.63.1.1-20.1999 10066829 PMC98955

[108] Plomp M, Carroll AM, Setlow P, Malkin AJ. 2014. Architecture and assembly of the *Bacillus subtilis* spore coat. PLoS One 9:e108560. doi:10.1371/journal.pone.0108560 25259857 PMC4178626

[109] Castro-Córdova P, Otto-Medina M, Montes-Bravo N, Brito-Silva C, Lacy DB, Paredes-Sabja D. 2023. Redistribution of the novel *Clostridioides difficile* spore adherence receptor e-cadherin by TcdA and TcdB increases spore binding to adherens junctions. Infect Immun 91:e0047622. doi:10.1128/iai.00476-22 36448839 PMC9872679

[110] Castro-Córdova P, Mora-Uribe P, Reyes-Ramírez R, Cofré-Araneda G, Orozco-Aguilar J, Brito-Silva C, Mendoza-León MJ, Kuehne SA, Minton NP, Pizarro-Guajardo M, Paredes-Sabja D. 2021. Entry of spores into intestinal epithelial cells contributes to recurrence of *Clostridioides difficile* infection. Nat Commun 12:1140. doi:10.1038/s41467-021-21355-5 33602902 PMC7893008

[111] Sonenshein AL. 2000. Control of sporulation initiation in *Bacillus subtilis*. Curr Opin Microbiol 3:561–566. doi:10.1016/S1369-5274(00)00141-7 11121774

[112] Perego M. 1998. Kinase–phosphatase competition regulates *Bacillus subtilis* development. Trends Microbiol 6:366–370. doi:10.1016/S0966-842X(98)01350-X 9778730

[113] Brunsing RL, La Clair C, Tang S, Chiang C, Hancock LE, Perego M, Hoch JA. 2005. Characterization of sporulation histidine kinases of *Bacillus anthracis*. J Bacteriol 187:6972–6981. doi:10.1128/JB.187.20.6972-6981.2005 16199567 PMC1251614

[114] Diaz AR, Core LJ, Jiang M, Morelli M, Chiang CH, Szurmant H, Perego M. 2012. *Bacillus subtilis* RapA phosphatase domain interaction with its substrate, phosphorylated Spo0F, and Its inhibitor, the PhrA peptide. J Bacteriol 194:1378–1388. doi:10.1128/JB.06747-11 22267516 PMC3294843

[115] Alves Feliciano C, Douché T, Giai Gianetto Q, Matondo M, Martin-Verstraete I, Dupuy B. 2019. CotL, a new morphogenetic spore coat protein of *Clostridium difficile*. Environ Microbiol 21:984–1003. doi:10.1111/1462-2920.14505 30556639

[116] Haldenwang WG. 1995. The sigma factors of *Bacillus subtilis*. Microbiol Rev 59:1–30. doi:10.1128/mr.59.1.1-30.1995 7708009 PMC239352

[117] Ramos-Silva P, Serrano M, Henriques AO. 2019. From root to tips: sporulation evolution and specialization in *Bacillus subtilis* and the intestinal pathogen *Clostridioides difficile*. Mol Biol Evol 36:2714–2736. doi:10.1093/molbev/msz175 31350897 PMC6878958

[118] McKenney PT, Eichenberger P. 2012. Dynamics of spore coat morphogenesis in *Bacillus subtilis*. Mol Microbiol 83:245–260. doi:10.1111/j.1365-2958.2011.07936.x 22171814 PMC3256263

[119] Setlow P. 2014. Germination of spores of *Bacillus species*: what we know and do not know. J Bacteriol 196:1297–1305. doi:10.1128/JB.01455-13 24488313 PMC3993344

[120] Baloh M, Sorg JA. 2022. *Clostridioides difficile* spore germination: initiation to DPA release. Curr Opin Microbiol 65:101–107. doi:10.1016/j.mib.2021.11.001 34808546 PMC8792321

[121] Paidhungat M, Setlow B, Driks A, Setlow P. 2000. Characterization of spores of *Bacillus subtilis* which lack dipicolinic acid. J Bacteriol 182:5505–5512. doi:10.1128/JB.182.19.5505-5512.2000 10986255 PMC110995

[122] Yamazaki K, Kawai Y, Inoue N, Shinano H. 1997. Influence of sporulation medium and divalent ions on the heat resistance of *Alicyclobacillus acidoterrestris* spores. Lett Appl Microbiol 25:153–156. doi:10.1046/j.1472-765x.1997.00194.x 9281865

[123] Edwards AN, McBride SM. 2016. Isolating and purifying *Clostridium difficile* spores. Methods Mol Biol 1476:117–128. doi:10.1007/978-1-4939-6361-4_9 27507337 PMC5017156

