# Supplementary material for: Distinctive spore architecture and developmental biology of Turicibacter sanguinis reveal unexpected diversity among gut spore formers
Source: J Bacteriol. 2026 Jul 14;208(8):e00029-26. doi: 10.1128/jb.00029-26 (PMC13492147; doi:10.1128/jb.00029-26)
Supplement: Supplementary file 1 [file jb.00029-26-s0001.docx]

**C**

**B**

**A**

Figure S1. Supporting phylogenetic trees for the RP-concatenated sets of proteins. (A) Maximum Likelihood (ML) inference tree using sequences available from genomes of *Turicibacter* spp. (Table S2B). Outgroups sequences were obtained from representative genomes selected of Erysipelotrichales (NCBI:txid526525). (B) Maximum Likelihood (ML) and (C) Neighbour-joining (NJ) inference tree using 7 Ribosomal protein families (RP7_tree: L3, L14, L16, L22, L24, S3, S8) present in all genomes and outgroups in the analysis (Table S2B).

**A**

**B**

Figure S2. Comparison of *T. sanguinis* K031 cell morphotypes in YCFA broth versus YCFA agar plate. *T. sanguinis* K031 samples were collected from YCFA broth (A) or YCFA plates (B), after 8, 10, 12, 16, 24 or 48 h of incubation at 37 ºC in anaerobic conditions. Samples were stained with Hoechst and FM 4-64 and examined by phase contrast and fluorescence microscopy. Bar scale represents 10 µm.

Figure S3. Transmission electron micrographs of *T. sanguinis* K031 thin and thick outer coat spores. *T. sanguinis* K031 thin and thick outer coat purified spores from two different batches of spores from YCFA agar plates. TEM have a magnification of 36,000x. Bar scale represents 200 nm.

Figure S4. *T. sanguinis* K031 quantitative analysis of ultrastructural layers. Histogram of normal distribution of inner coat length (A), cortex length (B), core diameter (C) and ridge height (D) were plotted using Prism 8 software and Modes were highlighted. (E) and (F) QQ plots show normal distribution of outer coat length from thin and thick spores respectively. Statistical analysis for normal distribution was performed using D'Agostino & Pearson test. Each green (thin) and coral (thick) circle represents the average length of one spore.

Figure S5. Correlation analysis between whole spore size and spore core or layers length. Correlation analysis between whole spore diameter and Crust-like (A), Outer coat (B), Inner coat (C), Cortex (D) lengths, Core diameter (E) or Ridges height (F). Data were plotted using Prism 8 software. Statistical analysis was performed by two-tailed Pearson correlation analysis with a confidence interval of 95 %. R squared of each analysis was highlighted. Each circle symbol represents the average length of one spore. Green and coral circles represent thin and thick outer coat spores respectively.

**A**

**B**

**Figure S6. Pipeline of sporulation and germination orthologs identification in *T. sanguinis* K031 genome.**

The pipeline used for ortholog search consists of 2 steps represented in A and B panels. (**A**) The first step was to create a query list of sporulation and germination of query proteins using hallmark proteins of sporulation [onset, regulation of sigma factors, engulfment mechanism, feeding tube system], germination [germinant receptors, DPA channels and spore cortex lytic enzymes]. Proteins were extracted from most studied spore-former species (*B. subtilis, B. cereus, B. anthracis, C. perfringens* and *C. difficile)* and the final query list was tabulated as was described in panel A. (**B**) The second step consisted in the identification of domains and family in the query database (Table S4) and in all CDS found in *T. sanguinis* K031 genome through KEGG, COG and pfam databases. The identification of query orthologs in the *T. sanguinis* K031 genome was performed by manual search of domains/family from the query database in table S5, creating a list of sporulation and germination hits (Table S6).

**C**

**A**

**B**

Figure S7. Comparative analysis of the genetic context of signatures of the onset of sporulation of *T. sanguinis* K031 and well-studied spore-formers models. (A-C) Comparison of the genetic architecture of *sigH* (A), *sigA* (B) and *spo0A* (C) loci between Bacillales (*Bacillus subtilis* strain 168, *B. cereus* ATCC14579, *B. anthracis* AA), Clostridiales (*C. perfringens* SM101, *C. difficile* strain 630) and *T. sanguinis* K031. Homologous genes arrows are filled with the same arrow color except for the genes which have no homologs in this context, which arrows are colored white. (A) *sigH* loci from spore-forming models from Bacillales and Clostridiales share gene context with *sigH* from *T. sanguinis* K031 since each genome encode *rpmG*, *secE*, *nusG*, *rplK* and *rplA* downstream, and *rlmB* upstream of *sigH*. (B) *sigA* loci from *B. subtilis* strain 168 shows that only *B. subtilis* strain 168 share gene context with *sigA* loci from *T. sanguinis* J3478_13700 since *recO*, *era* and *cdd* are encoded upstream of *sigA*. The rest of *sigA* orthologs (J3478_14895, J3478_09755) in *T. sanguinis* K031 and *C. difficile* strain 630 do not share gene context. (C) *spo0A* loci from spore-forming models Bacillales and *C. perfringens* SM101 share gene context with *spo0A* loci from *T. sanguinis* since upstream of *spo0A* are encoded *recN* and *spoIVB*. Light green arrows represent hypothetical proteins (HP). The scale of 1Kb represent only *spo0A*, *sigH* and *sigA* sequences, the rest is a representation and is not necessarily illustrated to scale. Reverse complementary (RC) sequences are highlighted.

Figure S8. Comparative analysis of the genetic context of *sigF* of *T. sanguinis* K031 and well-studied spore-formers models. Comparison of the genetic architecture of *sigF* loci between Bacillales (*Bacillus subtilis* strain 168, *B. cereus* ATCC14579, *B. anthracis* AA), Clostridiales (*C. perfringens* SM101, *C. difficile* strain 630) and *T. sanguinis* K031. The *sigF* loci from spore-forming models share gene context with *sigF* from *T. sanguinis* K031 since *sigF* it is surrounded by *spoIIAA-AB* and operon *spoVA*. Light green arrows represent hypothetical proteins (HP). The scale of 1Kb represents only the *sigF* sequence, the rest is a representation and is not necessarily illustrated to scale. Reverse complementary (RC) sequences are highlighted.

**A**

**B**

Figure S9. Comparative analysis of the genetic context of *sigG*, *sigE*, and genes that regulate activation and activity of *sigE,* *spoIIGA* and *spoIIR* of *T. sanguinis* K031 and well-studied spore-formers models.(A-B) Comparison of the genetic architecture of the *spoIIGA, sigE, sigG* (A) and *spoIIR* (B) loci between Bacillales (*Bacillus subtilis* strain 168, *B. cereus* ATCC14579, *B. anthracis* AA), Clostridiales (*C. perfringens* SM101, *C. difficile* strain 630) and *T. sanguinis* K031. Homologous genes arrows are filled with the same color except for the genes which have no homologs in this context, which are colored white. Reverse complementary (RC) sequences are highlighted. (A) *sigG*, *sigE* and *spoIIGA* loci from all spore-forming models, Bacillales and Clostridiales share gene context with *sigG*, *sigE* and *spoIIGA* loci from *T. sanguinis* K031, *sigE* is encoded immediately upstream *sigG*, and *spoIIGA* is encoded immediately upstream *sigE*. Besides, like Bacillales, *ftsZ* and *ftsA* are encoded upstream near to *spoIIGA*, and *ileS* is encoded downstream near to *sigG*. (B) *spoIIR* loci from spore-forming models Bacillales and *C. perfringens* SM101 share gene context with *spoIIR* loci from *T. sanguinis* K031 since upstream of *spoIIR* are encoded *prmC* and *prfA*. Light green arrows represent hypothetical proteins (HP). The scale of 1Kb represent only *sigE*, *sigG*, *spoIIGA* and *spoIIR* sequences, the rest is a representation and is not necessarily illustrated to scale.

**Figure S10. Comparative analysis of the genetic context of *sigK* of *T. sanguinis* K031 and well-studied spore-formers models.** Comparison of the genetic architecture of the *sigK* loci between Bacillales (*Bacillus subtilis* strain 168, *B. cereus* ATCC14579, *B. anthracis* AA), Clostridiales (*C. perfringens* SM101, *C. difficile* strain 630) and *T. sanguinis* K031. Homologous genes arrows are filled with the same color except for the genes which have no homologs in this context, which are colored white. Reverse complementary (RC) sequences are highlighted. *sigK* loci from spore-forming models *B. cereus* ATCC14579 and *B. anthracis* AA share gene context with *sigK* from *T. sanguinis* K031, however, seems to be a genomic rearrangement because *sigK* of *T. sanguinis* present a change of direction in the reading frame respect to Bacillales before mentioned, and *nadD*, that in Bacillales it is downstream of *sigK* in the same direction of the reading frame, and in *T. sanguinis* it is downstream but in the opposite direction. Light green arrows represent hypothetical proteins (HP). The scale of 1Kb represent only *sigK* sequence, the rest is a representation and is not necessarily illustrated to scale.

**A**

**B**

Figure S11. Comparative analysis of the genetic context of genes that regulate activation and activity of *sigF* of *T. sanguinis* K031 and well-studied spore-formers models. (A-B) Comparison of the genetic architecture of the *spoIIAA, spoIIAB* (A) and *spoIIE* (B) loci between Bacillales (*Bacillus subtilis* strain 168, *B. cereus* ATCC14579, *B. anthracis* AA), Clostridiales (*C. perfringens* SM101, *C. difficile* strain 630) and *T. sanguinis* K031. Homologous genes arrows are filled with the same color except for the genes which have no homologs in this context, which are colored white. Reverse complementary (RC) sequences are highlighted. (A) *spoIIAA* and *spoIIAB* loci from spore-forming models Bacillales and Clostridiales share gene context with *spoIIAA* and *spoIIAB* from *T. sanguinis* since both are encoded together, and *sigF* is encoded downstream of *spoIIAB* in each genome. (B) *spoIIE* loci from spore-forming models *B. cereus* ATCC14579, *B. anthracis* AA and *C. perfringens* SM101 share gene context with *spoIIE* from *T. sanguinis* since downstream of *spoIIE* it is encoded *tilS* and *hpt*. Light green arrows represent hypothetical proteins (HP). The scale of 1Kb represents only *spoIIAA*, *spoIIAB* and *spoIIE* sequences, the rest is a representation and is not necessarily illustrated to scale.

**A**

**B**

**C**

Figure S12. Comparative analysis of the genetic context of genes that regulate activation and activity of *sigG* of *T. sanguinis* K031 and well-studied spore-formers models. (A-C) Comparison of the genetic architecture of the *spoIIQ* (A), *spoIIIAC-AD-AE-AH* (B) and *gerM* (C) loci between Bacillales (*Bacillus subtilis* strain 168, *B. cereus* ATCC14579, *B. anthracis* AA), Clostridiales (*C. perfringens* SM101, *C. difficile* strain 630) and *T. sanguinis* K031. Homologous genes arrows are filled with the same color except for the genes which have no homologs in this context, which are colored white. Reverse complementary (RC) sequences are highlighted. (A) *spoIIQ* loci from spore-forming models *B. cereus* ATCC14579 and *B. anthracis* AA share gene context with *spoIIQ* from *T. sanguinis* K031 since upstream near of *spoIIQ* is encoded *spoIID*. (B) *spoIIIAC*, *spoIIIAD*, *spoIIIAE* and *spoIIIAH* loci from spore-forming models Bacillales and Clostridiales share gene context with *spoIIIAC*, *spoIIIAD*, *spoIIIAE* and *spoIIIAH* from *T. sanguinis* K031 since upstream, near of the beginning of the operon, is encoded *epf* (except in *C. difficile* strain 630). (C) *gerM* loci from spore-forming models Bacillales share gene context with *gerM* loci from *T. sanguinis* K031 since upstream and near of *gerM* is encoded *gerE*, and like in *B. subtilis* strain 168 genome, is encoded downstream *rph*. *gerM* is represented by peach arrows. Light green arrows represent hypothetical proteins (HP). The scale of 1Kb represent only *spoIIQ*, *spoIIIAA-AH* and *gerM* sequences, the rest is a representation and is not necessarily illustrated to scale.

**A**

**B**

**C**

Figure S13. Comparative analysis of the genetic context of genes that regulate activation and activity of *sigK* of *T. sanguinis* K031 and well-studied spore-formers models. (A-C) Comparison of the genetic architecture of the *spoIVB* (A), *spoIVFB* (B) and *bofA* (C) loci between Bacillales (*Bacillus subtilis* strain 168, *B. cereus* ATCC14579, *B. anthracis* AA), Clostridiales (*C. perfringens* SM101, *C. difficile* strain 630) and *T. sanguinis* K031. Homologous genes arrows are filled with the same color except for the genes which have no homologs in this context, which are colored white. Reverse complementary (RC) sequences are highlighted. (A) *spoIVB* loci from spore-forming models Bacillales and *C. perfringens* SM10 share gene context with *spoIVB* loci from *T. sanguinis* K031 since upstream and downstream of *spoIVB* are encoded *recN* and *spo0A*. (B) *spoIVFB* loci from spore-forming models Bacillales share gene context with *spoIVFB* loci from *T. sanguinis* K031 since upstream of *spoIVFB* are encoded *mreC* and *mreD* and downstream are encoded *rplU*, *rpmA* and *obgE*. (C) *bofA* loci from spore-forming models Bacillales share gene context with *bofA* loci from *T. sanguinis* K031 since upstream of *bofA* are encoded *recR* and *dnaX*. Light green arrows represent hypothetical proteins (HP). The scale of 1Kb represents only *spoIVB*, *spoIVFB* and *bofA* sequences, the rest is a representation and is not necessarily illustrated to scale.

**A**

**B**

**C**

Figure S14. Comparative analysis of the genetic context of engulfment related proteins of *T. sanguinis* K031 and well-studied spore-formers models. (A-C) Comparison of the genetic architecture of the *spoIID* (A), *spoIIM* (B) and *spoIIP* (C) loci between Bacillales (*Bacillus subtilis* strain 168, *B. cereus* ATCC14579, *B. anthracis* AA), Clostridiales (*C. perfringens* SM101, *C. difficile* strain 630) and *T. sanguinis* K031. Homologous genes arrows are filled with the same color except for the genes which have no homologs in this context, which are colored white. Reverse complementary (RC) sequences are highlighted. (A) *spoIID* loci from spore-forming models *Bacillus subtilis* strain 168 and *C. perfringens* SM101 share gene context with *spoIID* loci from *T. sanguinis* K031 since upstream to *spoIID* are encoded *atpC* and *atpD*. (B) *spoIIM* loci from *C. perfringens* SM101 share gene context with *spoIIM* loci from *T. sanguinis* K031 since upstream of *spoIIM* are encoded *spoIVB* and *spo0A*. Otherwise, downstream of *spoIIM* from *T. sanguinis* K031 are encoded *xerD* and *deoB*, like in *B. cereus* ATCC14579 and *B. anthracis* AA. (C) *spoIIP* loci from spore-forming models *Bacillus subtilis* strain 168 and *C. difficile* strain 630 share gene context with *spoIIP* loci from *T. sanguinis* K031 since downstream of *spoIIP* is encoded *lepA*. Light green arrows represent hypothetical proteins (HP). The scale of 1Kb represents only *spoIID*, *spoIIM* and *spoIIP* sequences, the rest is a representation and is not necessarily illustrated to scale.

**A**

**B**

Figure S15. Comparative analysis of the genomic context of transcriptional factors feed forward loops during sporulation of *T. sanguinis* K031 and well-studied spore-formers models. (A-B) Comparison of the genetic architecture of the *gerE* (A) and *spoVT* (B) loci between Bacillales (*Bacillus subtilis* strain 168, *B. cereus* ATCC14579, *B. anthracis* AA), Clostridiales (*C. perfringens* SM101, *C. difficile* strain 630) and *T. sanguinis* K031. Homologous genes arrows are filled with the same color except for the genes which have no homologs in this context, which are colored white. Reverse complementary (RC) sequences are highlighted. (A) *gerE* loci from spore-forming models Bacillales share gene context with *gerE* from *T. sanguinis* K031 since each genome encode *gerM* downstream of *gerE*. (B) *spoVT* loci from spore-forming models Bacillales and Clostridiales share gene context with *spoVT* loci from *T. sanguinis* since upstream of *spoVT* it is encoded *mfd*. Light green arrows represent hypothetical proteins (HP). The scale of 1Kb represents only *gerE* and *spoVT* sequences, the rest is a representation and is not necessarily illustrated to scale.

Figure S16. Comparative analysis of the genetic context of putative germination receptor *gerAA-AB-AC* and *gerKA-KB-KC* of *T. sanguinis* K031 and well-studied spore-formers models. Comparison of the genetic architecture of *gerAA-AB-AC* and *gerKA-KB-KC* loci between Bacillales (*Bacillus subtilis* strain 168, *B. cereus* ATCC14579, *B. anthracis* AA), Clostridiales (*C. perfringens* SM101, *C. difficile* strain 630) and *T. sanguinis* K031. Reverse complementary (RC) sequences are highlighted. *gerA* and *gerK* loci from Bacillales and *C. perfringens* SM101 do not share genetic context with *gerA* and *gerK* loci from *T. sanguinis* K031, however they are in the same way encoded next to each other. Light green arrows represent hypothetical proteins (HP). The scale of 1Kb represent only *gerA* and *gerK* sequences, the rest is a representation and is not necessarily illustrated to scale.

**A**

**B**

Figure S17. Comparative analysis of the genetic context of germination spore channel related proteins of *T. sanguinis* K031 and well-studied spore-formers models. (A-B) Comparison of the genetic architecture of the *gerQ* (A) and *spoVA* operon (B) loci between Bacillales (*Bacillus subtilis* strain 168, *B. cereus* ATCC14579, *B. anthracis* AA), Clostridiales (*C. perfringens* SM101, *C. difficile* strain 630) and *T. sanguinis* K031. Homologous genes arrows are filled with the same color except for the genes which have no homologs in this context, which are colored white. Reverse complementary (RC) sequences are highlighted. (A) *gerQ* share gene context with spore-forming models *B. cereus* ATCC14579 and *B. anthracis* AA, since upstream to *gerQ* are encoded *cwlJ* and *pta* (light peach and yellow arrows respectively). (B) *spoVAC-VAD-VAE* loci from all spore-forming models Bacillales and Clostridiales, and *spoVAF* just in Bacillales, share gene context with *spoVA* operon loci from *T. sanguinis* K031 since are encoded together as operon and upstream is encoded *sigF*. Light green arrows represent hypothetical proteins (HP). The scale of 1Kb represents only *gerQ* and *spoVAC-VAF* sequences, the rest is a representation and is not necessarily illustrated to scale.

**A**

**A**

**B**

**C**

**D**

Figure S18. Comparative analysis of the genetic context of germination lytic enzymes *cwlJ* and *sleM* and the adjuvants Gpr and GerF of *T. sanguinis* K031 and well-studied spore-formers models. (A-D) Comparison of the genetic architecture of the *gerF* (A), *cwlJ* (B), *sleM* (C) and *gpr* (D) loci between Bacillales (*Bacillus subtilis* strain 168, *B. cereus* ATCC14579, *B. anthracis* AA), Clostridiales (*C. perfringens* SM101, *C. difficile* strain 630) and *T. sanguinis* K031. Homologous genes arrows are filled with the same color except for the genes which have no homologs in this context, which are colored white. Reverse complementary (RC) sequences are highlighted. (A) *gerF* or *lgt* loci from spore-forming models *Bacillus subtilis* strain 168 and *B. anthracis* AA GBAA_5391 share gene context with *gerF* loci from *T. sanguinis* K031 since upstream is encoded *hprK* and downstream *ppaX*. Besides, share *uvrA* and *trxB* with *B. anthracis* AA genome. (B) *cwlJ* loci from spore-forming models *B. cereus* ATCC14579 and *B. anthracis* AA share gene context *cwlJ* loci from *T. sanguinis* K031 genome since upstream *cwlJ* is encoded *pta* and downstream *gerQ*. (C) *sleM* loci from *C. perfringens* SM101 do not share gene context with *sleM* loci from *T. sanguinis* K031. (D) *gpr* loci from spore-forming models Bacillales and *C. difficile* strain 630 share gene context with *gpr* loci from *T. sanguinis* K031 since upstream and downstream is encoded *rpsT* and *lepA*. Also, like in *B. subtilis* strain 168, *spoIIP* was found encoded immediately downstream of *gpr*. Light green arrows represent hypothetical proteins (HP). The scale of 1Kb represent only *cwlJ* and *gpr* sequences, the rest is a representation and is not necessarily illustrated to scale.
